# NMR-Based Configurational Assignments of Natural Products: Gibbs Sampling and Bayesian Inference Using Floating Chirality Distance Geometry Calculations

**DOI:** 10.3390/md20010014

**Published:** 2021-12-22

**Authors:** Stefan Immel, Matthias Köck, Michael Reggelin

**Affiliations:** 1 Clemens-Schöpf-Institut für Organische Chemie und Biochemie, Technische Universität Darmstadt, Alarich-Weiss-Straße 4, 64287 Darmstadt, Germany; 2 Alfred-Wegener-Institut für Polar-und Meeresforschung in der Helmholtz-Gemeinschaft, Am Handelshafen 12, 27570 Bremerhaven, Germany; mkoeck@awi.de

**Keywords:** distance geometry calculations, configurational analysis, chirality, NMR spectroscopy, residual dipolar couplings, assignment probabilities, statistical error analysis

## Abstract

Floating chirality restrained distance geometry (fc-rDG) calculations are used to directly evolve structures from NMR data such as NOE-derived intramolecular distances or anisotropic residual dipolar couplings (RDCs). In contrast to evaluating pre-calculated structures against NMR restraints, multiple configurations (diastereomers) and conformations are generated automatically within the experimental limits. In this report, we show that the “unphysical” rDG pseudo energies defined from NMR violations bear statistical significance, which allows assigning probabilities to configurational assignments made that are fully compatible with the method of Bayesian inference. These “diastereomeric differentiabilities” then even become almost independent of the actual values of the force constants used to model the restraints originating from NOE or RDC data.

## 1. Introduction

The determination of the relative or even absolute configuration of natural products has a long-standing history in organic chemistry. Despite huge progress in modern CASE (computer-assisted structure elucidation) algorithms [1,2,3,4,5,6,7,8,9,10,11,12,13], there is still no fully automated standard protocol available that solves all issues [14,15,16] and the “growing and general problem of structural mischaracterization” [17,18,19,20,21]. Given the known constitution of compounds, NMR-derived parameters such as scalar couplings and cross-relaxation (NOE or ROE)-derived interproton distances [22,23] add valuable information to the relative configuration and conformation of compounds. In addition to these traditional NMR parameters measured in isotropic solutions, auxiliary information can be obtained from residual dipolar or quadrupolar couplings (RDCs [24,25,26,27,28] and RQCs [24,29,30,31]), as well as residual chemical shift anisotropies (RCSAs [3,6,32,33,34,35,36,37,38]) measured in anisotropic environments of single or multi-alignment media [24,39,40,41,42,43,44,45,46]. These anisotropic parameters contain additional valuable angle information between even distant molecular fragments, which turn out to be very helpful with regard to the exact molecular geometry.

The elucidation of molecular configurations and/or conformations from NMR data can be divided into two major categories as either “static” or “dynamic” approaches. In the “static” approximation, pre-calculated molecular models are tested against experimental NMR data and are then selected or discarded via quality-of-fit parameters [2,5,7]. Relative molecular energies can be considered in this process, though it is known that Boltzmann-type averaging might be misleading when force-field (FF) or density functional theory (DFT) energies computed for isolated molecules are used [47].

The more advanced “dynamic” procedures consider molecular flexibility and allow conformations (or even configurations) to dynamically respond to restraints set by the experimental NMR data [48]. In this context, restrained molecular dynamics (rMD) [49,50,51] simulations, MD calculations with tensorial orientational constraints (MDOC) [52,53,54,55,56] on RDCs, and other simulation techniques have been used [57,58,59,60]. In some of these approaches, the alignment medium (AM) itself has been incorporated in atomistic simulations.

Most of the above approaches intrinsically rely on force-fields and are accordingly strongly biased towards low-energy structures, tending to overlook correct high-energy diastereomers (for a prominent example, see the particularly strained *trans*-annulated ring configuration of palau’amine [19,61,62]). Still, in combinations with FF methods, the problem of high-energy barriers between diastereomers (inversions of configurations) has to be overcome. Other solutions suggested to the problem of structure elucidation imply very crude alignment models [63,64]. In a recent paper, Thiele et al. [65,66] aim at a semi-analytical approach towards configurational assignments based on RDCs using spherical harmonics and redundant internal coordinates. However, this approach suffers from the requirement of very large RDC data sets (including long-range couplings) in five or typically more AMs. In addition, this approach is quite sensitive to missing elements in the RDC matrix, which is associated with the problem that not all required RDCs may be measurable in all alignment media.

In this context, we recently have revived the rather old, but powerful, method of distance geometry (DG) [67,68,69,70,71] calculations to incorporate NOE [72,73,74,75] as well as RDC [62,76,77] restraints to tackle the configurational problem. DG simulations are intrinsically free of physical force-field parameters and allow simultaneous use of variable sources of NMR-derived restraints, including RDCs obtained from multi-alignment media investigations, which is, with the exception of TITANIA [65,66], not possible with other methods [52,53,54,55,56].

**Scheme 1:**
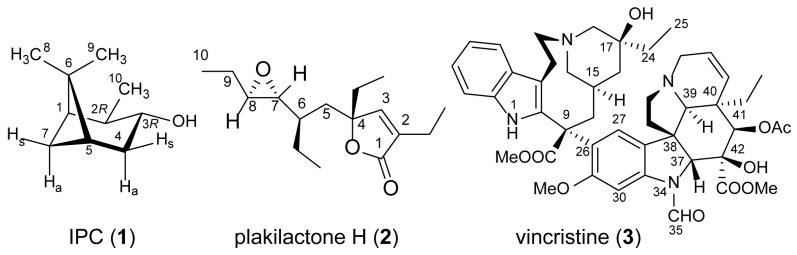
Formulas, atom labeling, and numbering of **1-3**.

The DG formulation of the configurational problem is relatively straightforward (for details see below), and only one type of empirical parameters is used within the DG framework. Most importantly, these are the so-called force constants that are used to incorporate and scale NOE and, e.g., RDC restraints. Consequently, we would like to explore the implications of varying the relative force constants in rDG simulations in this report in detail. In particular, their implications for the resulting quantitative differentiability of diastereomers will be demonstrated for three examples of natural compounds (Scheme 1), namely isopinocampheol (IPC, **1**), plakilactone H (**2**) (Table S2), and vincristine (**3**) (Table S3).

## 2. Results and Discussion

The general static procedure for the configurational and conformational analysis of unknown natural products is to compare experimental NMR parameters $X^{exp}$ with back-calculated values $X^{calc}$ for pre-computed molecular models of all possible diastereomers. Quality-of-fit indicators or error functions such as summed weighted squared deviations $\chi^{2}$ (Equation (1)), Pearson correlation coefficients R or $R^{2}$, or *Q*-factors [78] (Equation (2)) can be used, while different weighting factors $w_{i}$ for the experimental data (e.g., RDCs i) may be considered if required.

(1) $$\chi^{2}=\sum^{\;}w_{i}{\left(X_{i}^{exp}-X_{i}^{calc}\right)}^{2}=\sum^{\;}w_{i}\;\Delta X_{i}^{2}$$

(2) $$Q=\sqrt{\frac{\sum^{\;}w_{i}{\left(X_{i}^{exp}-X_{i}^{calc}\right)}^{2}}{\sum^{\;}w_{i}{\left(X_{i}^{exp}\right)}^{2}}}$$

Common practice is then to plot, e.g., *Q*-factors for all diastereomers as bar graphs and to accept the lowest sum of squared violations $\chi^{2}$ or *Q*-value as an indication for the “correct” configuration and/or conformation. However, these qualitative assessments do not yet make quantitative statements on diastereomeric differentiabilities [79].

A first approach to quantify estimates of the certainty with which configurational assignments can be made from anisotropic NMR data such as RDCs has been proposed using the Akaike information criterion (*AIC*) [80] as the error function (Equation (3)) [2,5,7,79,81].

(3) $$AIC=2k+\underset{i}{\sum }\frac{{\left(X_{i}^{exp}-X_{i}^{calc}\right)}^{2}}{\sigma_{X_{i}}^{2}}$$

Here, $\sigma_{X_{i}}^{2}$ represents the squared standard deviations of the parameters $X_{i}$, and k is an additional penalty denoting the number of fitting parameters used for back-calculating $X^{calc}$. Since k is constant in all considerations outlined in this report (e.g., k=5 for all 5-parameter RDC alignment-tensor fits), it can be dropped from all further considerations here.

For independent observables $X_{i}$ with Gaussian-distributed multiplicative probabilities (likelihoods) $p_{i}\;\propto \exp\left(-1/2\;\Delta X_{i}^{2}/\sigma_{X_{i}}^{2}\right)$, the sum $AIC\propto -\log(\prod^{\;}p_{i})\propto \sum^{\;}\Delta X_{i}^{2}/\sigma_{X_{i}}^{2}$ represents a log-likelihood function, which should become minimal for the best-fit molecular configuration assigned therefrom. What will become crucial in the sequel is that the conditional probability P(D|θ) that the experimental data “D” measured for a given (pre-computed) molecular structure “θ” is then described by the following likelihood function omitting only a constant normalization factor:

(4) $$P\left(D|\theta \right)\propto \underset{i}{\prod }\exp\left(-\frac{1}{2}\cdot \frac{\Delta X_{i}^{2}}{\sigma_{X_{i}}^{2}}\right)=\exp\left(-\frac{1}{2}\cdot \underset{i}{\sum }\frac{\Delta X_{i}^{2}}{\sigma_{X_{i}}^{2}}\right)=\exp\left(-\frac{1}{2}\cdot AIC\right).$$

The certainty or relative probability with which two diastereomers A and B can be differentiated is then given by their relative AIC weights or the ratio $P_{A}/P_{B}$ by the following:

(5) $$\frac{P_{A}}{P_{B}}=e^{\left(-\frac{1}{2}\left(AIC_{A}-AIC_{B}\right)\right)}=e^{\left(-\frac{1}{2}\Delta AIC_{A-B}\right)}.$$

The main issue with this approach is that the uncertainties $\sigma_{X_{i}}$ in Equations (3) and (4) are unknown, and that these not even need to be equal for all experimental NMR parameters available (hence the index i). Their values can be estimated only roughly from experimental data ($\sigma_{exp}$), and usually all other sources of uncertainties are neglected. Especially, these include uncertainties in alignment tensors [82] and the singular value decomposition (SVD) [83] used for back-calculating the RDC data ($\sigma_{calc}$), or thermal motions (vibrations) of “real” molecules rather than “static” molecular models ($\sigma_{vibr}$) [79,84]. As the propagation of Gaussian-distributed errors adds up the squared standard deviations, the total uncertainties in Equation (3) become $\sigma_{total}^{2}=\sigma_{exp}^{2}+\sigma_{calc}^{2}+\sigma_{vibr}^{2}$. In a recent report, we have shown that with $\sigma_{vibr}^{2}\gg \sigma_{exp}^{2}+\sigma_{calc}^{2}$, thermal vibrations are by far the most important source of uncertainty and almost exclusively determine the power (or weakness!) of the AIC procedure to discriminate between alternative diastereomers based on, e.g., pre-computed DFT models [84]. In any case, this approach then requires quantum chemical frequency calculations of all structure models under consideration, which quickly becomes prohibitively expensive when evaluating even moderately sized and flexible analytes. Because of the unknown uncertainties $\sigma_{X_{i}}$, how to simultaneously consider different NMR parameters (e.g., NOEs and/or RDCs) in the context of AIC scores and Equation (3) also remains ambiguous.

### 2.1. NMR Restraints in Distance Geometry Calculations

In a fundamentally different approach, we have proposed that NMR data should not be evaluated against pre-calculated molecular models, but that these models should evolve automatically from NMR parameters [62,76,77]. With floating chirality (fc) [85,86,87] restrained distance geometry (rDG) [70] and distance-bounds-driven dynamics (DDD) [68,88] calculations, configurations, and conformations change dynamically, and thus all possible diastereomers emerge for any given molecular constitution (which must be known). Here, holonomic distance restraints derived from 1,2-(bonds), 1,3-(angles), 1,4-connectivities (torsions), and optional chiral volumes (signed vector triple products typically used for, but not limited to, sp^3^- and sp^2^-type atomic centers), as well as NMR parameters, set the limits for an automated sequence of short simulated annealing pseudo-MD simulations in 4D and 3D space, from which molecular structures are sampled. In practice, in fc-rDG/DDD simulations chiral volume restraints are applied only to sp^2^-centers to keep them planar ($V_{chir}=0$) as well as to an arbitrarily chosen single stereogenic element in order to avoid enantiomeric structures.

It is important to note that this procedure does not rely on any conventional (physical) force-field (FF), any other parameters, or any pre-calculated structures. Only a single (possibly low-quality) FF- or DFT-derived molecular model of arbitrary configuration is required to automatically set up the distance bounds (i.e., the atomic distance matrix) based on molecular connectivity. The whole subsequent process of structure elucidation has been proven to be independent of this first guess and is free of any intrinsic bias towards specific diastereomers (usually $2^{N-1}$ structures for N stereogenic centers) including the one used as input.

The fc-rDG/DDD approach uses a dimensionless total “pseudo energy” penalty function $E_{total}$, where distance (holonomic bond lengths) errors ($E_{dist}$), chiral volume violations ($E_{chir}$), and deviations of experimental NMR parameters such as NOE distances ($E_{NOE}$), RDCs ($E_{RDC}$), and others (RQCs, RCSAs, etc.) are all summed up (Equations (6)–(8)).

(6) $$E_{total}=E_{dist}+E_{chir}+E_{NOE}+E_{RDC}+\ldots$$

(7) $$E_{RDC}=\frac{1}{2}K_{RDC}\underset{RDCs}{\sum }{\left(D_{i}^{exp}-D_{i}^{calc}\right)}^{2}$$

(8) $$E_{NOE}=\frac{1}{2}K_{NOE}\underset{NOEs}{\sum }\ln^{2}\left(d^{exp}/d^{calc}\right)$$

All these pseudo energy terms take the form of harmonic sums of squared violations ${\left(\Delta X\right)}^{2}={\left(X_{i}^{exp}-X_{i}^{calc}\right)}^{2}$, except for NOEs, for which a log-normal potential (Equation (8)) is suited better (for details see below). Here, the $K_{X}$ are force constants that are chosen empirically in the first place for each of the pseudo energy terms, and they can be considered as weighting factors for different types of experimental NMR data. Note that these energy terms $E_{X}$ should not be confused with “real” molecular energies. Nevertheless, the negative partial derivatives −∂E/∂r with respect to 4D or 3D Cartesian coordinates (i.e., the negative Cartesian gradients of E) are considered as forces that drive the structure evolution from NMR data in this approach.

**Figure 1:**
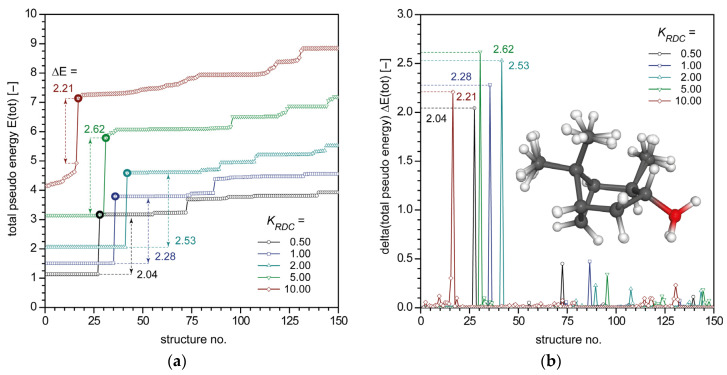
(**a**) Plot of the total “pseudo energy” of ranked rDG structures of IPC (**1**) showing the first 150 out of 1000 structures generated using a three AM RDC data set with varying force constants $K_{RDC}$. All structures within the first (lowest) energy plateau display the correct stereochemical assignment for IPC; the first wrong configuration (wrong C-2 diastereomer) in the ranked sequence is marked by bold circles. (**b**) Plot of energy changes $E_{n+1}-E_{n}$ for subsequent energy-sorted rDG structures of IPC using different force constants on RDCs (“first derivative” of plot (**a**)). The peak heights show the energy jumps between alternate configurational families and are labelled accordingly in plot (**a**). The inset molecular models in plot (**b**) show a superposition of all five best-fit lowest energy structures of IPC with almost identical geometries, but with the typical daisy-flower-like appearance of undefined methyl groups as free rotors.

As a prototypical example, Figure 1 shows the results of sampling molecular structures of IPC (**1**) (Figure S1) from a fc-rDG/DDD simulation using 11 D1CH RDCs measured in three alignment media (AM) each. All RDC violations are summed up in the rDG total pseudo energy ($E_{total}$) (cf. Equations (6) and (7)), which is then plotted for all structures sampled as a function of their rank in energy-sorted lists (Figure 1a). Alternatively, Figure 1b displays the energy difference $\Delta E=E_{N}-E_{N-1}$ between successive structures (quasi-first derivatives of plots in Figure 1a); both graphs are shown for variations of the harmonic force constant $K_{RDC}$ that has been employed for the rDG simulations. Alternate configurational families of IPC are clearly indicated in the former plot by energy steps and by peaks in the latter. Here, the correlated configurations of C-1 and C-5 were fixed by chiral volume restraints in order to avoid enantiomeric structures. In all cases, the lowest pseudo energy plateau corresponds to the correct relative configuration of IPC, including correct assignments of the diastereotopic protons or methyl groups at C-4, C-6, and C-7 (which is formally equivalent to assigning the configuration of a stereogenic center). The first wrong stereochemical assignment (inverted configuration of C-2) is separated by a significant energy step therefrom, and the differentiability of the correct configuration of IPC from its diastereomers is obvious.

To add an important side note, rDG simulations also do not require vibrational corrections, e.g., for RDCs, as the entire setup is designed to produce (vibrationally) averaged molecular geometries. During the DDD simulated annealing, this implies tensor SVD fits (for each AM individually) between the experimental ($D^{exp}$) and back-calculated ($D^{calc}$) RDCs at each time-step to update the forces. Here, the final structures and the back-calculated RDCs automatically fulfil the least-squares fit minimum boundary condition for deriving the components of the Saupe tensor S as defined by Equation (9) with zero total thermal corrections.

(9) $$\frac{\partial E_{RDC}}{\partial S_{\alpha \beta }}=\frac{1}{2}K_{RDC}\cdot \frac{\partial \sum^{\;}{\left(D^{exp}-D^{calc}\right)}^{2}}{\partial S_{\alpha \beta }}=0$$

This boundary condition does not hold when just evaluating pre-computed structures against RDC data, unless vibrational corrections to both RDCs and the components of the alignment tensor are applied [84].

However, the size of the energy steps or the corresponding height of the peaks (Figure 1), as well as the number of correct structures sampled (horizontal shift of steps and peaks), depends on the force constant applied on the RDCs. At first glance, only a qualitative estimate of the certainty of configurational assignments seems possible from the plots in Figure 1a,b, with their reliability increasing the more extended the plateaus become and the higher the energy steps $\Delta E_{total}$ are (differences in NMR data violations).

### 2.2. Bayesian Inference from RDC-Driven rDG Calculations

**Figure 2:**
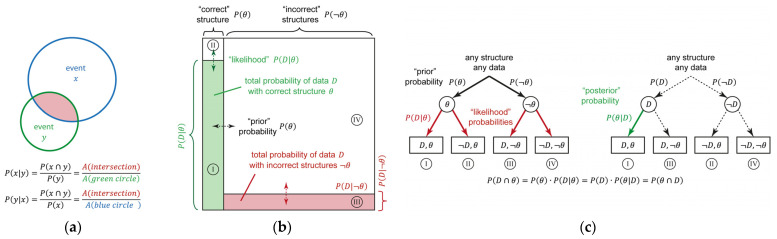
(**a**) Illustration of conditional probabilities from intersecting areas: Given two sets of overlapping events x and y, the conditional probability P(x|y) of an event x given that y has happened equals the probability of x and y happening together, divided by the probability of y. The analogous definition of P(y|x) leads to P(x|y)≠P(y|x). (**b**) Visualization of Bayes’ theorem: Given are the prior (horizontal axis) probability P(θ) of the correct structure and P(¬θ) for the entire ensemble of all alternative incorrect structures (“¬θ ” means “not correct”, with P(θ)+P(¬θ)=1 ). With a high likelihood (conditional probability on the vertical axis) that the data D matches the correct structure P(D|θ) (“true positives”), and a low likelihood that the experimental data D match an incorrect structure P(D|¬θ) (“false positives”), the total posterior probability P(θ|D) (note the swap of conditions!) of the correct structure given the observed NMR data set becomes the green area, divided by the sum of the green plus the red area. The denominator in Equation (10) then matches the constant probability P(D) that the data were observed in the first place, leading to Equation (11) as used in the main text. (**c**) Visualization of two decision trees with inverted order: the prior probability is marked by the solid black arrows, the model likelihoods by red arrows, and the sought posterior probability P(θ|D) is indicated by the green arrow; unknown probabilities are marked by dashed arrows. Bayes’ theorem then follows immediately from the definition of conditional probabilities as depicted in (**a**) and the equivalence shown. The roman numerals designate the corresponding areas and probabilities in plot (**b**).

The most rigorous and stringent statistical way to quantify the reliability of models based on experimental data is Bayesian inference [89,90,91,92,93,94,95,96,97]. In fact, in a beautiful review, Habeck et al. have shown that “the determination of (…) structures from experimental data is an ill-posed inverse problem”, and that “the only way to quantify uncertainty systematically and consistently is through probabilities” [96]. Bayesian inference has been used for conformer generation [98,99,100] and to analyze protein RDCs [101,102,103], as well as in the field of NMR crystallography [104,105,106], but has been ignored in the context of configurational assignments of small molecules. The entire problem of structure determination can be traced back to the conditional probability P(θ|D), which must be read as a probability P that “during the experiment the molecular structure was θ”, given the result that the “data D was recorded” [96] (see Figure 2).

(10) $$P\left(\theta |D\right)=\frac{A\left(green\right)}{A\left(green\right)+A\left(red\right)}=\frac{P\left(\theta \right)P(D|\theta )}{P\left(\theta \right)P(D|\theta )+P\left(\neg \theta \right)P(D|\neg \theta )}.$$

In Habeck’s notation, this is expanded to P(θ|D,I) to quantify the plausibility of a structure θ (here including the question of configuration and conformation) in the context of the experimental data “D”and information “I” [96]. In our case, this information I is the constitution of a compound, which must be known prior to rDG simulations. In fact, all probabilities discussed below should be regarded as conditional probabilities “given I has happened”, but for convenience, we drop “I” from the formulas below.

In statistics, Bayes’ theorem [107] (cf. Equation (11)) is perhaps the most important formula in probability and the holy grail in data science, very much like the fully automated configurational analysis in chemistry [5,24]. It inverts the sought-after, but difficult to determine, probability P(θ|D) to the accessible quantity P(D|θ) (for a visualization of the discussion following below, see Figure 2).

(11) $$P\left(\theta |D\right)=\frac{1}{P\left(D\right)}\cdot P\left(\theta \right)\cdot P(D|\theta )$$

Here, the probability P(D|θ) is the so-called likelihood function, which relates theory to experiment. Reading this conditional probability as “how likely is the experimental data D, given that the structure was indeed θ”, it becomes clear that this is related to the AIC score—which in fact is a log-likelihood function—and Equation (4) as described above. Equations (6)–(8) represent the full Hamiltonian from which the fc-rDG/DDD structures evolve, and the corresponding Boltzmann weight given by Equation (12) becomes the likelihood function to be considered here:

(12) $$P\left(D|\theta \right)\propto \exp\left(-\beta E_{total}\left(\theta \right)\right),$$

where β is thermodynamically equivalent to, but not to be confused with an inverse temperature. With Equation (7), this becomes the following for the RDC part:

(13) $$P\left(D|\theta \right)\propto \exp\left(-\beta \frac{1}{2}\cdot \underset{i}{\sum }K_{X}\;\Delta X_{i}^{2}\right).$$

Both Equations (4) and (13) imply Gaussian-shaped likelihood functions that become identical for β=1 and $\sigma_{X}=1/\sqrt{K_{X}}$. In methods that combine physical force-fields with NMR restraints, β can be considered as a weighting factor for the experimental data [108]. However, in rDG there are no such force-fields, and β can be set to unity, as it simply would modify the force constants $K_{x}$ (for a detailed discussion, see below). Also note that rDG-derived pseudo energies are always dimensionless quantities, and though $E_{total}$ should not be confused with a physical molecular energy, it nevertheless carries statistical significance, because of which rDG structures are sampled in this approach.

The second quantity P(θ) in Equation (11) represents a naturally occurring prior probability that reflects previous knowledge about the system before NMR experiments. In fc-rDG/DDD simulations, there is no bias towards any specific configuration or conformation based on physical force-field energies, and actually all structures generated by the rDG simulation occur with uniform prior probability P(θ):

(14) $$P\left(\theta \right)=1/N_{DG},$$

where $N_{DG}$ is just the total number of structures generated in the entire rDG ensemble of all structures. In statistics, this unbiased prior probability is frequently called an “uninformative prior”.

The last remaining term P(D) on the right side of Equation (11) is just another constant normalization factor—namely, the probability that the data D were measured at all—that can be discarded from all considerations following. With this, combining Equations (11)–(14) yields the desired probability P(θ|D):

(15) $$P\left(\theta |D\right)\propto \frac{1}{N_{DG}}\cdot \exp\left(-E_{total}\left(\theta \right)\right).$$

Now, all considerations above can be expanded to include multi-alignment media RDC data sets applied as simultaneous rDG restraints. Moreover, this extension can also include different NMR parameters, such as NOEs, and the force constants (weighting factors) used for different data sets need not be equal. Re-normalization (“marginalization”) of Equation (15) then directly results in the following:

(16) $$P\left(\theta |D\right)=\frac{1}{Z}\cdot \exp\left(-E_{total}\left(\theta \right)\right),$$

where the re-normalization factor $Z=\sum^{\;}\exp\left(-E_{total}\right)$ is computed from the entire canonical ensemble of structures generated by the rDG simulation (i.e., integration over the entire curves presented in Figure 1a). The probability P(θ|D) is then called the posterior probability, which reflects everything known about the structure, based on the experimental NMR data D that was actually measured.

The above considerations show that the entire rDG approach and this Boltzmann-weighted type of Gibbs sampling of molecular configurations and/or conformations is indeed fully compatible with the laws of thermodynamics and Bayesian inference based on the statistical interpretation of $E_{total}$ presented here. In retrospect, the rDG distance bounds and NMR restraints fully define the Hamiltonian of the system under consideration. If the rDG energy were to be a real energy—which it is obviously not, but there are no other force-field parameters in the rDG approach!—it then would be straightforward to agree that the thermodynamically correct Boltzmann-type averaging justified from Bayesian inference is the only natural way to compute averages.

The total Bayesian probability that the configuration of the compound under investigation was indeed “Θ” can then be computed by the following Equation (17).

(17) $$P\left(\Theta |D\right)=\frac{1}{Z}\cdot \underset{\Theta }{\sum }\exp\left(-E_{total}\left(\theta_{i}\right)\right)$$

Here, the summation runs over all individual molecular structures $\theta_{1\cdots N_{DG}}$ generated by a rDG simulation that have a specific configuration “Θ” but can adopt arbitrary conformations. The proper normalization factor Z is then defined as described above. Indeed, Habeck has proposed that “any structure determination problem” should be computed from this Bayesian probability, and that this process should be properly termed “inferential structure determination (ISD)” [96]. For clarity and convenience, we have defined the probability with which a given configuration “Θ” of an unknown compound can be deduced from the NMR data available, the “diastereomeric differentiability” (dd) of Θ:

(18) dd(Θ)≡P(Θ|D).

### 2.3. RDC-Driven rDG Calculations of IPC (1)

**Figure 3:**
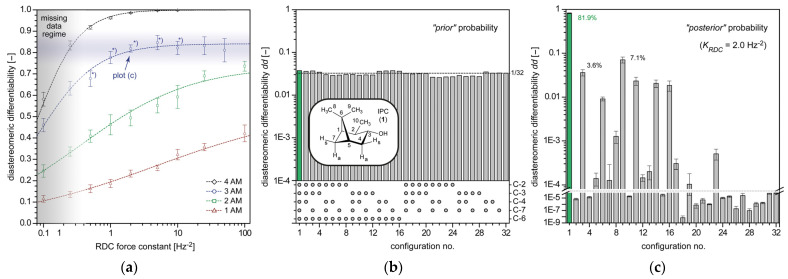
(**a**) Plot of the diastereomeric differentiability of the correct stereochemical assignment of IPC (including all diastereotopic groups) as a function of the number of different alignment-media RDC data sets used (1–4 AM) and the force constants $K_{RDC}$ (in [Hz^−2^]) employed for the rDG simulations. For each of the data points marked by asterisks, a corresponding single “energy step” plot is shown in Figure 1a,b. Data and error bars were obtained from 10 separate rDG runs per point and 1000 structures per rDG run, and the RDC data was taken from Ref. [76]. The gray shaded area on the left shows “weak restraints” and therefore defines a missing data regime for which ($K_{RDC}\to 0$ Hz^−2^) the dd values must converge to 1/32 for IPC (see next plot). (**b**) Plot of sampling probabilities of different configurations of IPC (**1**) derived from floating chirality rDG simulations without NMR restraints. The different configurations of the stereogenic centers C-2 and C-3, as well as all possible arrangements of diastereotopic groups (methylene groups C-4, C-7, and methyl groups at C-6), are sampled with almost uniform probabilities; the dashed black line gives the statistical average for 32 alternate configurations (dd=1/32 ). The dots in the punch card style plot below the bar chart indicate configurations identical to the correct assignment of IPC for the various carbon atoms. (**c**) Plot of distinct assignment probabilities (diastereomeric differentiabilities) of all 32 IPC configurations for the point marked in plot (**a**) by an arrow, with $K_{RDC}=2.0$ Hz^−2^, and three AM RDC data sets; the ordering of configurations is identical to Figure 3b. The correct configuration of IPC is identified with ≈82% certainty (green bar), followed by ≈7% probability of the C-2 epimer (configuration #9) and ≈3–4% probability for the alternate assignment of the diastereotopic protons of the C-4 methylene group (configuration #3); note the logarithmic scale on the ordinate, as for better comparability, the scale was chosen to be identical for the plots (**b**,**c**). In Bayesian statistics, plot (**b**) shows the prior probability $P\left(\Theta_{j}\right)$, and plot (**c**) corresponds to the posterior probability $P\left(\Theta_{j}|D,I\right)$ of configurational assignments before and after acquisition of the NMR data, respectively.

In order to explain the effect of the “diastereomeric differentiability” calculations, the fc-rDG/DDD simulations of IPC (**1**) (Table S1) already mentioned above shall be taken up again here. Figure 3a shows dd values of the correct configuration of IPC—including the correct assignment of all diastereotopic groups, which is formally equivalent to assigning configurations of stereogenic centers—over all alternative assignments. The data are plotted as a function of the number of RDC alignment data sets combined (colored curves with M=1−4 AM used), and as a function of the force constant $K_{RDC}$ applied during the rDG simulations, respectively. Here, the data points marked by asterisks on the blue curve (3 AM RDC data sets) correspond exactly to the data shown in Figure 1, and the maximal probability for the correct stereochemical assignment of IPC using three AM RDC data sets is computed to about dd≈80%. This value increases to dd>95% when using four AM RDC data sets (black curve) but is significantly lower when using 1-2 RDC data sets only.

In Figure 3b,c, typical probability histograms are plotted for sampling all alternate stereochemical assignments of IPC from these rDG calculations. The plot in Figure 3b shows the sampling probability in the absence of any NMR restraints, based solely on holonomic restraints (distance bounds $E_{dist}$ and chiral volume restraints $E_{chir}$) used for encoding the molecular constitution of IPC. The four diastereomers of IPC (C-2 and C-3 stereogenic centers), as well as all alternate arrangements of the diastereotopic protons in methylene groups (C-4 and C-7) and methyl groups at C-6, are sampled with almost uniform probability and with uniform total pseudo energy $E_{total}$ (32 structures in total, all with $\Delta E<10^{-3}$). Minor deviations from a perfect flat distribution can be seen for the *cis*- and *trans*-arrangement of the substituents at C-2 and C-3, as the density of states must not necessarily be exactly equal for all diastereomers.

In Bayesian statistics, Figure 3b corresponds to the “uninformed” prior probability $P\left(\Theta_{j}\right)$ of all j=1⋯32 configurations of IPC that are to be considered before NMR data is acquired. A similar example is also provided in the SI of Ref. [62] for the more complex structure of axinellamine A (eight stereogenic centers resulting in 128 diastereomers).

In contrast, Figure 3c shows the posterior probability distribution $P\left(\Theta_{j}|D,I\right)$ of configurational assignments. It is this distribution (note the logarithmic scale!) that emerges from the rDG simulations and Equations (17) and (18), and it reflects the updated configurational information after the NMR data “D” (three AM) was recorded. It thus can be used directly to quantify the probability (certainty) with which the configuration of IPC can be assigned, given that the molecular constitution (information “I”) of the analyte is known.

As a convenient alternative to Equation (11), the Bayesian prior and posterior assignment certainties plotted in Figure 3b,c can be rationalized not only in terms of probabilities, but also in terms of odds (ratios of probabilities for correct and incorrect assignments) [109]:

(19) $$O\left(\Theta_{j}|D\right)=O\left(\Theta_{j}\right)\cdot \frac{P\left(D{|\Theta }_{j}\right)}{P\left(D|\neg \Theta_{j}\right)}.$$

Here, the prior odds (before NMR data are measured) of assigning the correct configuration of an analyte are denoted by $O\left(\Theta_{j}\right)$, which reflects the ratio of correct: incorrect stereochemical assignments. For IPC, this equals $O\left(\Theta_{j}\right)=P\left(\Theta_{j}\right):P\left(\neg \Theta_{j}\right)=1:31$. The factor $P\left(D{|\Theta }_{j}\right)/P\left(D|\neg \Theta_{j}\right)$ in Equation (19) is called the Bayes factor [110], reflecting the likelihoods that the measured data matches the correct (Θ) or incorrect (¬Θ, i.e., “not Θ”) configuration. In other words, the Bayes factor is the ratio of “true positives” vs. “false positives”, with the experimental data identifying either the “correct” or “incorrect” configuration. The Bayes factor must literally be read as an update factor that quantifies the change in assignment probabilities brought about by the experimental NMR data.

For any stereochemical structure elucidation, it is desirable to achieve posterior odds (after NMR measurements) of e.g., $O\left(\Theta_{j}|D\right)>95:5$ in favor of a correct assignment. Thus, for IPC (**1**), Equation (19) commands rather large Bayes factors ≳600 to assure configurational assignments at high confidence levels. It is exactly this factor in combination with the misconception of likelihood functions P(D|θ) vs. posterior (conditional) probabilities P(θ|D), as well as the biased method of structure generation through FF or DFT methods, that in the scientific literature is frequently misinterpreted, leading to overestimated differentiabilities of diastereomers based on RDC data [2,5,7].

**Figure 4:**
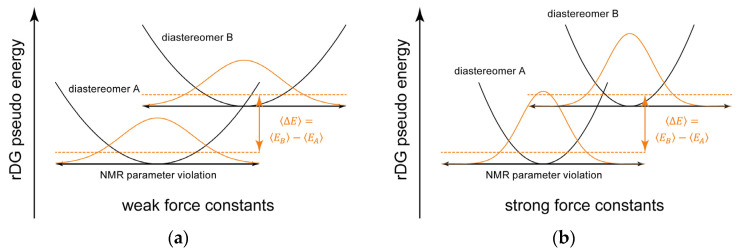
Schematic representation of sampling structures of two diastereomers, A and B, from harmonic potentials (black curves) with weak (**a**) or strong (**b**) force constants on NMR parameter violations and the corresponding likelihood functions (orange curves). The (averaged) expectation values (i.e., the integrals $\langle E\rangle =\int^{\;}E\left(X\right)p\left(X\right)dX$) and the rDG energy differences $\langle \Delta E\rangle =\langle E_{B}\rangle -\langle E_{A}\rangle$ (dashed horizontal orange lines) then become independent of the force constants applied.

Another important conclusion that can be drawn from Figure 3a is that for sufficient experimental NMR data available (>3 AM) and sufficiently strong weighting of the RDC restraints ($K_{RDC}>1$ Hz^−2^), the dd values computed for IPC converge to constant values, though the corresponding individual curves of the pseudo energy plots shown in Figure 1 look different. Most notably, this dd value becomes independent of the actual value of the force constant applied in the rDG calculations over a very large range $K_{RDC}>1-100$ Hz^−2^ spanning two orders of magnitude. This is obviously due to compensating effects originating from the definition of the rDG pseudo force-field (Equations (6) and (7)) and the Bayesian likelihood function (Equations (4) and (12)): setting tighter restraints on RDCs (trying to lower $E_{RDC}$) increases violation energies in bond lengths ($E_{dist}$) and chiral volumes ($E_{chir}$), and vice versa. Simultaneously, increasing the force constant increases the energy steps as presented in Figure 1a, but it also decreases the sampling efficiency (number of correct structures generated) of rDG simulations and increases the error bars (Figure 3a). However, the integrated assignment probabilities (cf. Equation (17)) then become essentially independent of the force constants. To put it in other words, sampling from harmonic potentials $1/2\;K\Delta X^{2}$ with Boltzmann-type acceptance ratios $\propto \exp\left(-1/2\;K\Delta X^{2}\right)$ must yield results based on $\langle E_{DG}\rangle$ that do not depend on the actual value of K (see Figure 4).

Now, it is precisely this relation between the force constants $K_{X}$ (Equation (13)) and the corresponding standard deviations $\sigma_{X}$ (Equation (4))—in statistics, the latter are called nuisance parameters [96,101,103]—that gives the rDG calculations an invaluable advantage over the AIC-based probability derivations. Instead of having to estimate the unknown model uncertainties or standard deviations (which are dominated by thermal vibrations [84]) in the AIC approach, the rDG force constants implicitly set the limits on these uncertainties from configurational sampling. The stiffer the chosen force constant in rDG, the narrower the corresponding probability densities become for sampling structures from NMR data, and vice versa (see Figure 4).

As a side note, we would like to mention that the consistent decrease in the dd values on the left side of Figure 3a ($K_{RDC}<0.25$ Hz^−2^) is just indicative for very weak NMR restraints. For $K_{RDC}\to 0$ Hz^−2^, it inevitably must follow also that $E_{RDC}\to 0$, and consequently, the dd values must finally approach a flat, almost uniform distribution (uninformed prior probability), as displayed by Figure 3b with dd→1/32 for IPC. All simulations shown in Figure 3a have used exactly the same simulation parameters (DDD total simulation lengths and time steps, etc.), except for the number of RDC restraints and the force constant applied. These simulations become numerically unstable if $K_{RDC}$ is increased even further and if the DDD integration time steps are not lowered accordingly (which we intentionally did not do here), so due to practical considerations, the maximum value of $K_{RDC}$ is limited to the reasonable range as displayed.

### 2.4. rDG Calculations Using Combined NOE and RDC Restraints

In our rDG approach, NOE/ROE and RDC restraints can be applied simultaneously to the problem of configurational assignments. As NMR parameter deviations are multiplicative to probabilities but are additive to the rDG pseudo energy penalty function (Equations (6)–(8)), the Boltzmann-type weighting scheme defined by Equations (12) and (16) can also be applied to the combined use of NOEs and RDCs, even though the individual force constants may differ.

As uncertainties in NOE-derived distances are usually in the order of Δd≈0.1−0.5 Å and errors in RDCs are in the range of ΔD≈0.5−2.0 Hz, it seems natural to consider both restraints with different magnitudes or relative weights of the force constants in the order of $K_{NOE}/K_{RDC}\approx 10/1$ during the rDG simulations. In all previous reports on the rDG methodology [62,77], we have used similar harmonic potentials on both NOE and RDC parameters (cf. Equation (7)). This certainly applies well to signed RDCs, which can take either negative or positive values. However, for strictly positive NOE-derived distances, it has been shown that a logarithmic-harmonic (“log-normal”) likelihood function (cf. Equation (8)) is better suited to reproduce distributions of experimental errors [108,111].

**Figure 5:**
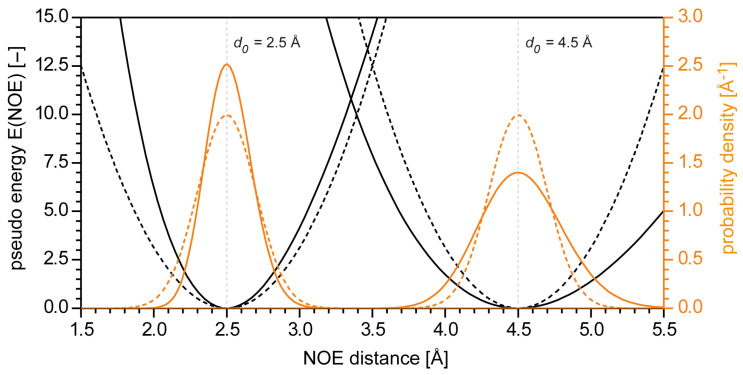
Plot of “log-harmonic” (solid black lines) and harmonic (dashed black lines) NOE restraint potentials (left ordinate) and the corresponding normalized likelihood (probability density) functions $\propto \exp\left(-E_{NOE}\right)$ (orange lines, right ordinate); curves are plotted for two different NOEs with $d_{0}=2.5$ Å and 4.5 Å, and using $K_{NOE}^{log-normal}=250.0$ and $K_{NOE}^{harmonic}=25.0$ Å^−2^, respectively. With $K_{NOE}^{log-normal}=d_{0}^{2}\cdot K_{NOE}^{harmonic}$, both types of potentials have the same curvature around $d_{0}$. The harmonic potential is symmetric about $d_{0}$, whereas the log-normal potentials are steeper (stiffer) for $d<d_{0}$, and softer (more flexible) for $d>d_{0}$.

Figure 5 shows that harmonic potentials (dashed lines) weigh NOE distance restraints with constant widths (uncertainties), whereas log-normal potentials (solid lines) are stiffer on short NOE-derived distances and more flexible on longer distances. Thus, the latter functional relationship is more natural, since large NOE distances are experimentally harder to measure and are subject to larger uncertainties. In addition, the curvature (stiffness) of both potentials (i.e., the second derivatives $\partial E_{NOE}^{2}/\partial^{2}d$) centered at a given NOE distance $d_{0}$ differ by a factor $1/d_{0}^{2}$, with the log-normal potentials being “softer” for larger distances. As typical NOE distances are in the range of $d_{0}\approx 2.0-5.0$ Å, the log-normal force constant $K_{NOE}$ should be chosen even an additional order of magnitude higher with $K_{NOE}/K_{RDC}\approx 100/1$. Also note that the log-normal type force constant $K_{NOE}$ becomes dimensionless. In practice, we choose force constants on RDCs in the range of $K_{RDC}\approx 0.1-2.0$ Hz^−2^ and on NOE distances $K_{NOE}\approx 25-250$.

#### 2.4.1. Plakilactone H (2)

**Figure 6:**
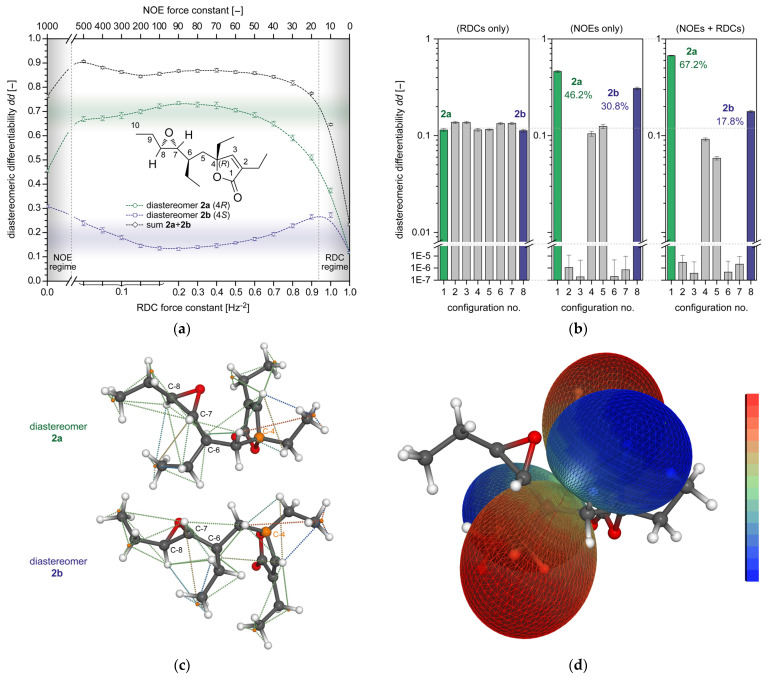
(**a**) Sampling probabilities for diastereomers of plakilactone H (**2**) as a function of the type of restraints applied and the magnitude of the force constants used in rDG simulations. The data set of 25 experimental NOEs taken from Ref. [112] and 13 RDCs is compatible with both C-4 epimers (diastereomers **2a**, blue curve, and **2b**, green curve) of plakilactone H (**2**). The Bayesian assignment probabilities for **2a** and **2b** are plotted as calculated from counter-variant variations (about three orders of magnitude) of the force constants $K_{NOE}$ (top abscissa) and $K_{RDC}$ (bottom abscissa) applied to the NOE and RDC restraints, respectively. (**b**) Plot of typical Bayesian posterior assignment probabilities using different combinations RDC or/and NOE restraints ($K_{NOE}=50$ and $K_{RDC}=0.5$ Hz^−2^). (**c**) Molecular models of the diastereomers **2a** and **2b** identified (C-4 epimers); the dotted lines indicate the NOE restraints, respectively. (**d**) Plot of a typical rDG-derived alignment tensor for the correct diastereomer **2a**. In this figure, all error bars were obtained from 10 separate rDG simulations per point (10,000 structures per rDG run); for details, see text.

In order to elucidate the effect of variations in the different (restraint-dependent) force constants on the probabilities of configurational assignments, we have chosen plakilactone H (**2**) as an illustrative example (Figure 6). The experimental NOEs measured for **2** were insufficient to fully derive the relative configuration of all four stereogenic centers simultaneously [112]. In our previous study [77], we also confirmed this fact by rDG simulations, which showed that the relative configuration of three out of four stereogenic centers (C-6, C-7, and C-8) can be deduced from the NOE restraints, but both C-4 epimers (diastereomers **2a** and **2b**) could not be assigned unequivocally, though we have not computed quantitative diastereomeric differentiabilities previously.

The inability of NOE data to resolve this structural problem is due to the high flexibility of the molecule and the fact that the NOEs mainly involve rotatable ethyl groups and unassigned diastereotopic protons of the corresponding methylene groups only, and this in particular hampers the assignment of the quaternary center C-4.

In order to evaluate the value of simultaneously applying NOEs and RDCs to this structural problem, and in the absence of experimental data, we have added an artificial RDC data set to the major diastereomer **2a** based on a randomly generated alignment tensor. This single-AM test set consisted of 13 D1CH RDCs involving four methine RDCs, five methylene groups (used as unassigned sums of two C-H RDCs), and four methyl RDCs.

The effects of applying both NOE and RDC restraints and simultaneously counter-variant changes of the force constants in the range of $K_{NOE}=0-1000$ and $K_{RDC}=1.0-0.1$ Hz^−2^ are plotted in Figure 6a. As expected, the differentiation between diastereomers **2a** over **2b** is increased by the introduction of RDCs, since the RDC data set was generated for a model of **2a**. However, surprising is the fact that the rDG-derived differentiability of **2a** (dd of 65–75%) and **2b** (15–25%) represented by the blue and green shaded areas in Figure 6a remains remarkably unaffected by changing the relative magnitude of the individual NOE and RDC force constants over a range spanning almost three orders of magnitude. This is a clear and decisive advantage of the rDG approach: it uses a minimum of prior information (only the correct molecular constitution and the corresponding bond lengths are required), and solely the NMR data drives the structure evolution. As discussed above, the compensating effects on the split terms of the pseudo energy $E_{NOE}$ and $E_{RDC}$ then ensure that the resulting configurational assignments become almost unaffected by the very sparse empirically chosen parameters (force constants) involved.

Only inappropriately chosen ratios $K_{NOE}/K_{RDC}$ significantly change the results. Extending Figure 6a to the left enters a NOE-dominated regime (grey shaded area), and the dd values for **2a** and **2b** drop to lower values. Similarly, towards the right edge of Figure 6a it can be seen that with $K_{NOE}/K_{RDC}\to 0$ ($K_{NOE}=0$ and $K_{RDC}=1.0$ Hz^−2^), a regime that is dominated by RDCs without using NOEs is entered (grey shading). Significantly, for the flexible structure of plakilactone H (**2**), the 13 RDC parameters used here cannot differentiate alone between different diastereomers, and the dd values of **2a** and **2b** both sharply drop to a random chance of ≈1/8 (four stereogenic centers, and thus eight diastereomers of **2**).

In addition, Figure 6b shows exemplary Bayesian posterior probabilities computed for the eight diastereomers of **2** using different NMR restraints (RDCs or NOEs) or combinations thereof. Solely using RDC restraints does not lead to a significant differentiation of any diastereomer of **2** above a random chance of 1/8. NOE restraints alone turn the decision in favor of diastereomer **2a** (**2a**:**2b** ≈ 46:31, configurations no. #1:#8), yet this certainty is increased further to **2a**:**2b** ≈ 67:18 by simultaneously applying NOE and RDC restraints to the fc-rDG/DDD analysis.

Arguably, one could add additional information to the configurational assignment problem of **2** by performing a presumably very time-consuming (FF- or DFT-based) configurational and conformational analysis, but this is exactly what we would like to avoid. Instead of adding bias based on in vacuo optimized structures (which quickly can become somehow arbitrary for flexible compounds [47], in particular if strong polar intramolecular interactions are present such as H-bonds etc. that can falsify the results), we want to use as few prior assumptions as possible, and we would like to evolve molecular structures solely through the NMR data itself. Then, the combination of quantitative NOEs and RDCs turns out to be an extremely powerful one, as both parameters average mathematically differently for alternate structure models.

#### 2.4.2. Vincristine (3)

**Figure 7:**
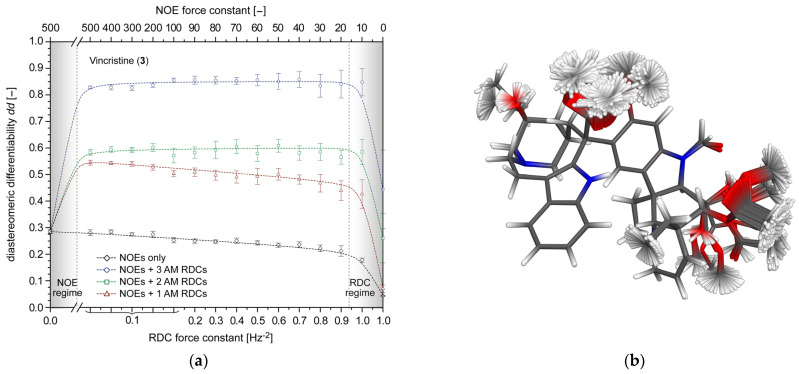
(**a**) Certainties of correct stereochemical assignment of vincristine (**3**) as a function of the force constants applied to NOEs (dimensionless $K_{NOE}$, top abscissa) and RDCs ($K_{RDC}$ in [Hz^−2^], bottom abscissa) during rDG simulations. Data and error bars were obtained from 10 separate rDG simulations per point (10,000 structures per rDG run), and the NMR data were taken from Refs. [62,76]. (**b**) Typical superposition of molecular structures for the correct configuration of vincristine (**3**) obtained from a fc-rDG/DDD calculation using NOE restraints and three AM RDC data sets.

Another example proving the independence of the rDG calculations from ad hoc assumptions or arbitrary chosen force constants is presented in Figure 7 for the alkaloid vincristine (**3**) [113,114]. For this compound with nine stereogenic centers, we applied a theoretical NMR data set of 23 NOEs and up to 3 · 24 RDCs (three AM) with unassigned methylene groups, which has been used also in Refs. [62,76].

Here, we have varied the corresponding rDG force constants in the range of $K_{RDC}=0.1-1.0$ Hz^−2^ and inversely $K_{NOE}=500-10$, and we have computed the diastereomeric differentiability of the correct configuration of **3** therefrom (using the NOE data and varying amounts of 1–3 RDC data sets).

In this analysis, we have intentionally left out the quaternary stereogenic center C-42, as there is no NMR data—neither NOEs nor RDCs—associated with its exocyclic substituents, and both epimers of C-42 actually turned out in a 1:1 ratio. Based on the data used, the configuration of **3** can be assigned with a certainty of dd≈85% (NOEs + 3 AM RDCs). The remaining uncertainty can be traced back mainly to the configuration of C-17, and the flexibility of the ethyl side chain attached to this stereogenic center. Decreasing the number of RDC data sets from alternate AM also decreases the reliability of the configurational assignment.

It is remarkable that in all cases depicted in Figure 7a, the certainty of the configurational assignments of **3** does not crucially depend on the ratio $K_{NOE}/K_{RDC}$ of force constants chosen but remains almost unaffected thereby over about two orders of magnitude. The size of the error bars given in Figure 7a increases slightly to the right, indicating some variability of the results obtained from 10 independent rDG simulations, but the mean values remain almost constant.

## 3. Methods

The mathematics of RDC calculations used here have been taken from Glaser et al. [115], and the formalism on how to include NOE and RDC data in 4D and 3D fc-rDG simulations as implemented in our software package ConArch^+^ has been described in full detail in Refs. [62,76,77].

An initial input structure is used by DG only for setting up the holonomic bounds and distance matrices (±1% bond lengths), and subsequent configurational and conformational sampling is carried out by the ConArch^+^/DG (Table S4) software package in an automated sequence of steps. First, molecular structures are generated in four-dimensional (4D) space (“metrization” step, i.e., embedding based on holonomic distance bounds), followed by a 4D “floating chirality” restrained DG (fc-rDG) and distance-bounds-driven dynamics (DDD) simulation (simulated annealing). After reduction of dimensionality, the simulated annealing is repeated in 3D space, and each simulation in 4D and 3D is concluded by a gradient-descent type optimization of structures against all restraints, minimizing the total pseudo energy $E_{total}$. In all dynamics and optimization calculations, the negative partial derivatives $-\partial E_{total}/\partial r_{\alpha }$ of all energy terms with respect to 4D and 3D Cartesian atomic coordinates (α∈x,y,z(,w) for all atoms) are interpreted and used as forces governing the evolution of the system. All derivatives are calculated analytically by ConArch^+^/DG. During each step of the rDG/DDD runs using RDCs, full updates of the Saupe or alignment tensors are computed based on a singular-value decomposition (SVD) algorithm.

All fc-rDG/DDD calculations used here employed time steps of τ=5 fs and simulations with 5000 steps at T=300 K, followed by an additional 5000 steps of cooling to T→0 K, in 4D and 3D space, respectively. Effective force constants on holonomic distances and chiral volumes were used as specified in the original DG version ($K_{dist}=2.0$ Å^−2^ and $K_{chir}=2.0$ Å^−6^), and force constants applied to NOEs ($K_{NOE}$) and RDCs ($K_{RDC}$) were varied as specified in the text. Each simulation was set up to produce 1000 (**1**) or 10,000 (**2**, **3**) structures, and error bars plotted in the Figures were obtained from 10 independent rDG simulations using different random seeds. All ConArch^+^/DG calculations are fully parallelizable with almost linear efficiency, and a typical simulation on **2** (including all 4D and 3D steps) generating 10,000 structures on a 40-core node (Intel(R) Xeon(R) CPU E5-2670 v2 @ 2.50GHz) takes approx. 10 min. wall time.

In this report, error bars (uncertainties) on diastereomeric differentiabilities were computed from 10 repeated and independent fc-rDG/DDD simulations. However, only after finishing this manuscript was a more time-saving method implemented in ConArch^+^, based on Metropolis Monte-Carlo simulations using the ensemble of all rDG structures (here: a total of $N_{DG}$ molecular structures comprising all different configurations and conformations) that have been generated in a single simulation. The analysis is initiated by randomly picking a rDG structure $\theta_{1}$. Subsequent structures $\theta_{n}$ (n>1) are also picked randomly but are accepted according to the standard Metropolis criterion only if the total rDG pseudo energy decreases $\left(\Delta E=E\left(\theta_{n}\right)-E\left(\theta_{n-1}\right)\leq 0\right)$, or if a random number r uniformly distributed in the interval [0.0, 1.0) is less than exp(−ΔE); otherwise, the previous state is retained. Using, e.g., 10,000 Metropolis chains of length $N_{DG}$ each, the averages as well as the corresponding uncertainties of weights of individual configurations can be estimated efficiently and quickly without the necessity of recomputing the entire rDG/DDD protocol. For sufficiently large rDG simulations ($N_{DG}≳1000$, the uncertainties scale with $\sqrt{N_{DG}}$), the average Metropolis Monte-Carlo weights quickly converge to the Bayesian configurational probabilities discussed throughout this report, as expected for a canonical ensemble of Boltzmann-weighted entities.

**Figure 8:**
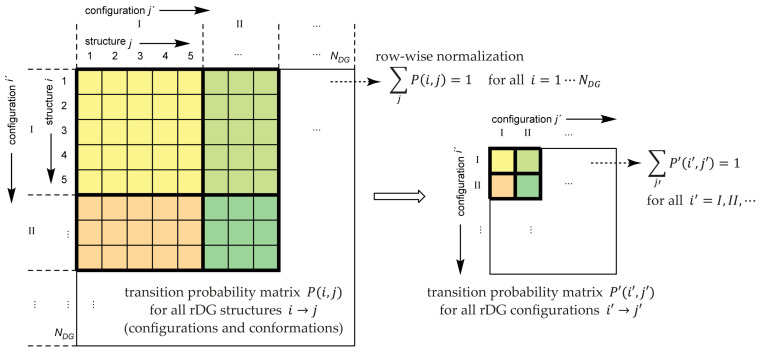
Scheme of contracting the Markov-chain transition probability matrix P(i,j) between all rDG structures i→j (with $i,j=1\cdots N_{DG}$, Arabic numerals) into a transition probability matrix $P{}^{\prime }\left(i{}^{\prime },j{}^{\prime }\right)$ between configurational families $i{}^{\prime }\to j{}^{\prime }$ (Roman numerals).

Similarly, all $N_{DG}$ molecular structures obtained from a rDG simulation can be used to construct a Markov-type process and a transition probability matrix P(i,j) between rDG structures i→j using the Metropolis criterion described above. This $N_{DG}\times N_{DG}$ square matrix can be contracted into a much smaller transition probability matrix $P{}^{\prime }\left(i{}^{\prime },j{}^{\prime }\right)$ between alternate configurations $i{}^{\prime }\to j{}^{\prime }$ by appropriate summation and re-normalization over all members for all configurational families (Figure 8).

Both matrices P and P’ are right-stochastic matrices with rows summing up to unity, and they feature probability (row) vectors π or $\pi {}^{\prime }$ that are stationary under application of the transition matrices (e.g., $\pi^{{}^{\prime }}P^{{}^{\prime }}=\pi {}^{\prime }$). Thus, the vectors π and $\pi {}^{\prime }$ are row eigenvectors of the probability matrices with eigenvalue 1. The Markov-chain steady-state probability distributions (averages) computed from these eigenvectors then correspond exactly to the Bayesian probabilities (differentiabilities) of rDG-derived structures or configurations, and the corresponding uncertainties (error bars) are estimated numerically as described above.

## 4. Conclusions

NOE- and RDC-driven restrained distance geometry (rDG) calculations represent a straightforward methodology to tackle the configurational assignment of structures with two or more stereogenic elements, including hitherto unknown natural compounds. For compounds with N stereogenic centers, there is no need to evaluate at least $2^{N-1}$ individual structures (each configuration may comprise many conformations) against NMR data, but one simulation allows for comprehensive configurational sampling, including assignments of diastereotopic atoms and groups if required. The rDG approach guarantees an unbiased sampling, and both the configuration and conformation of complex compounds can be established in a single simulation where structures evolve directly from the NMR data. The violations of the NMR restraints are described as DG pseudo energies using harmonic potentials on RDCs, and log-normals on NOEs. Though this pseudo energy is not to be confused with a “real” physical molecular energy, we have shown that it bears statistical significance and can be used to define probabilities to configurational assignments in full agreement with the method of Bayesian inference.

The determination of absolute configurations is impossible within the rDG framework, which is by definition inversion-invariant. However, once the correct relative configuration and conformation (note that rDG handles both issues!) of a given compound is known, this “posterior” information can be exploited easily to tackle the problem of absolute configurations using ECD or VCD calculations.

We have also demonstrated not only that the rDG-derived configurational assignments are a powerful approach to the interpretation of NMR data with high reliability, but that Bayesian “diastereomeric differentiabilities” are even independent over large ranges of absolute and relative values of weighting factors used in the rDG simulations to scale the experimental restraints. In addition, the method described allows arbitrarily combining restraints originating from different NMR parameters such as NOE or RDC data, including the possibility to simultaneously apply the latter in the context of multi-alignment media data sets.

## Data Availability

The full methodology outlined here for the interpretation of NOEs and RDCs has been implemented in our ConArch^+^ (Configurational Architect) program, which can be obtained along with the source code (free of charge for academic institutions) by request from our web site (https://www.chemie.tu-darmstadt.de/reggelin, accessed on 21 December 2021.)

## Supplementary Materials

The following are available online at https://www.mdpi.com/article/10.3390/md20010014/s1, Figure S1: RDC Data and Alignment Tensors of IPC (**1**), Table S1a,d: RDC Data for IPC (**1**), Table S2a,b: RDC and NOE Data for Plakilactone H (**2**), Table S3a,d: RDC and NOE Data for Vincristine (**3**), Table S4: Typical ConArch^+^/DG initialization parameters as used for all fc-rDG/DDD simulations in this report.
