# Supplementary material for: NMR-Based Configurational Assignments of Natural Products: Gibbs Sampling and Bayesian Inference Using Floating Chirality Distance Geometry Calculations
Source: Mar Drugs. 2021 Dec 22;20(1):14. doi: 10.3390/md20010014 (PMC8781118; doi:10.3390/md20010014)
Supplement: Supplementary file 1 [file marinedrugs-20-00014-s001.zip › marinedrugs-1501228-supplementary.pdf]

Supporting Information for

NMR-based Configurational Assignments of Natural Products: Gibbs Sampling and Bayesian Inference Using Floating Chirality Distance Geometry Calculations

Stefan Immel[a,] Matthias Köck[b], and Michael Reggelin[a]

[a] Clemens-Schöpf-Institut für Organische Chemie und Biochemie, Technische Universität Darmstadt, Alarich-Weiss-Straße 4, 64287 Darmstadt, Germany

[b] Alfred-Wegener-Institut für Polar- und Meeresforschung in der Helmholtz-Gemeinschaft, Am Handelshafen 12, 27570 Bremerhaven, Germany

Table of Contents

Formulas and Atom Numbering of Compounds 1, 2 and 3 1
RDC Data for IPC (1) 2
RDC and NOE Data for Plakilactone H (2) 5
RDC and NOE Data for Vincristine (3) 6

Formulas and Atom Numbering of Compounds 1, 2 and 3

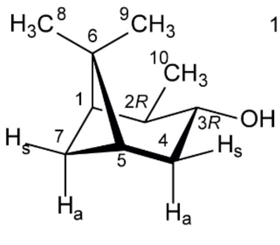

IPC (**1**)

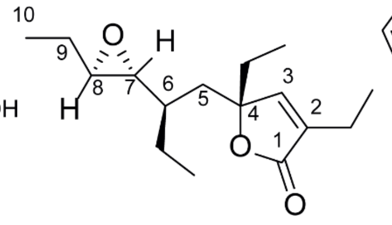

plakilalctone H (**2**)

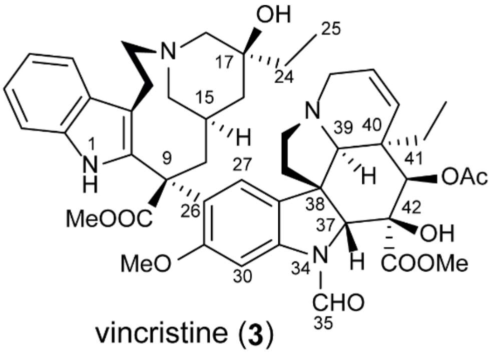

vincristine (**3**)

RDC Data for IPC (1)

The following Tables S1a-d and Figure S1 show the experimental and back-calculated RDC data used for the configurational analysis of IPC (**1**). The Tables were generated directly from the output files for the *ConArch*$^+$/*DG* best-fit (minimum pseudo energy) structure obtained from fc-rDG/DDD configurational and conformational analysis using experimental RDC data sets from four alignment media (K_{RDC} = 2.0 Hz^{-2}).

Table S1a. RDC data used for **1** (AM #1). The experimental data is listed as D_{exp}, and the RDCs back-calculated from the structure model are labeled D_{calc}; all values are given in [Hz]. In addition, the characteristics of the alignment tensor are listed.

```
SVD Best-Fit Saupe Vector S(zz), S(xx-yy), S(xy), S(xz), S(yz):
  1.513603e-03 -1.837005e-04 -1.176154e-04  2.295930e-04  5.777138e-04
Saupe Tensor (S):                                         Alignment Tensor (A):
 -8.486519e-04 -1.176154e-04  2.295930e-04                 -5.657680e-04 -7.841026e-05  1.530620e-04
 -1.176154e-04 -6.649514e-04  5.777138e-04                 -7.841026e-05 -4.433010e-04  3.851425e-04
  2.295930e-04  5.777138e-04  1.513603e-03                  1.530620e-04  3.851425e-04  1.009069e-03
Trace of Saupe Tensor:        0.000000e+00                Trace of Alignment Tensor:    0.000000e+00
Eigenvectors of Saupe Tensor (S):                         Eigenvectors of Alignment Tensor (A):
  6.457182e-01  7.596593e-01  7.723810e-02                  6.457182e-01  7.596593e-01  7.723810e-02
 -7.521820e-01  6.154113e-01  2.355657e-01                 -7.521820e-01  6.154113e-01  2.355657e-01
  1.314165e-01 -2.102062e-01  9.687843e-01                  1.314165e-01 -2.102062e-01  9.687843e-01
Eigenvalues of Saupe Tensor S(xx), S(yy), S(zz):          Eigenvalues of Alignment Tensor A(xx), A(yy), A(zz):
 -6.649178e-04 -1.007465e-03  1.672383e-03                 -4.432785e-04 -6.716432e-04  1.114922e-03

Alignment Tensor Irreducible Representation (A0, A1R, A1I, A2R, A2I):
  2.399562e-03  2.971891e-04  7.478027e-04 -1.188926e-04 -1.522434e-04

Tensor Properties:
A(axial)       =   1.672383e-03         # alignment tensor axial component   = 3/2*A(zz) = S(zz)
A(rhombic)     =   2.283647e-04         # alignment tensor rhombic component = A(xx) - A(yy) = 2/3*(S(xx) - S(yy))
A(rhombicity) =   1.365505e-01         # alignment tensor rhombicity        = A(rhombic) / A(axial)
A(asymmetry)   =   2.048258e-01         # alignment tensor asymmetry         = (A(xx) - A(yy))/A(zz) = (S(xx) - S(yy))/S(zz)
GDO            =   1.684036e-03         # generalized degree of order        = sqrt(3/2)*|A(xx),A(yy),A(zz)| = sqrt(2/3)*|S(xx),S(yy),S(zz)|

Results for Multi-Parameter SVD Fit of Calculated and Experimental Data:
           D(calc)[HZ]      +/- Error    D(exp)[HZ]      +/- Error  Rel. Weights           D(exp)-D(calc)        Normalized Weights    Atom Labels
D[01] =       6.671269              -      6.500000      0.500000      1.000000     r[01] =      -0.171269     w[01] =        0.090909 *) C1-H1
D[02] =       1.929977              -      1.800000      0.500000      1.000000     r[02] =      -0.129977     w[02] =        0.090909 *) C2-H2
D[03] =     -25.329241              -    -25.300000      0.500000      1.000000     r[03] =       0.029241     w[03] =        0.090909 *) C3-H3
D[04] =     -11.049904              -    -11.050000      0.500000      1.000000     r[04] =      -0.000096     w[04] =        0.090909 *) C4-H4s
D[05] =       7.527950              -      7.300000      0.500000      1.000000     r[05] =      -0.227950     w[05] =        0.090909 *) C4-H4a
D[06] =       6.156943              -      6.000000      0.500000      1.000000     r[06] =      -0.156943     w[06] =        0.090909 *) C5-H5
D[07] =     -21.767408              -    -21.950000      0.500000      1.000000     r[07] =      -0.182592     w[07] =        0.090909 *) C7-H7s
D[08] =      18.405904              -     18.450000      0.500000      1.000000     r[08] =       0.044096     w[08] =        0.090909 *) C7-H7a
D[09] =       0.162958              -      0.460000      0.500000      1.000000     r[09] =       0.297042     w[09] =        0.090909    C6-C8
D[10] =      -2.185509              -     -2.140000      0.500000      1.000000     r[10] =       0.045509     w[10] =        0.090909    C6-C9
D[11] =      -0.514035              -     -0.670000      0.500000      1.000000     r[11] =      -0.155965     w[11] =        0.090909    C2-C10

Results for Multi-Parameter Fit of Calculated and Experimental Data:
rank   =              5                 # rank of cosine matrix (check input if rank < 5)
cond   =   3.855250e+00                 # condition number of cosine matrix (check input and singular values if very large)
chisq =       0.024872                 # weighted total sum of squared residuals
aic    =      11.094353                 # information criterion (AIC) for 5 degrees of freedom
rmsd   =       0.157708                 # unweighted root mean square deviation
qfac   =       0.012596                 # weighted Q-Factor as defined by Cornilescu
r^2    =       0.999838                 # coefficient of determination r^2 = 1 - chi^2 / (weighted sum of squares)
```

Table S1b. RDC data used for **1** (AM #2). The experimental data is listed as D_{exp}, and the RDCs back-calculated from the structure model are labeled D_{calc}; all values are given in [Hz]. In addition, the characteristics of the alignment tensor are listed.

```
SVD Best-Fit Saupe Vector S(zz), S(xx-yy), S(xy), S(xz), S(yz):
  6.390761e-04 -1.214396e-03 -6.457956e-05 -1.355954e-04  3.964746e-04
Saupe Tensor (S):                                         Alignment Tensor (A):
 -9.267362e-04 -6.457956e-05 -1.355954e-04                 -6.178241e-04 -4.305304e-05 -9.039696e-05
 -6.457956e-05  2.876601e-04  3.964746e-04                 -4.305304e-05  1.917734e-04  2.643164e-04
 -1.355954e-04  3.964746e-04  6.390761e-04                 -9.039696e-05  2.643164e-04  4.260507e-04
Trace of Saupe Tensor:        0.000000e+00                Trace of Alignment Tensor:    0.000000e+00
Eigenvectors of Saupe Tensor (S):                         Eigenvectors of Alignment Tensor (A):
 -2.016793e-02  8.084779e-02  9.965224e-01                 -2.016793e-02  8.084779e-02  9.965224e-01
 -8.399167e-01 -5.420445e-01  2.697754e-02                 -8.399167e-01 -5.420445e-01  2.697754e-02
  5.423405e-01 -8.364517e-01  7.883732e-02                  5.423405e-01 -8.364517e-01  7.883732e-02
Eigenvalues of Saupe Tensor S(xx), S(yy), S(zz):          Eigenvalues of Alignment Tensor A(xx), A(yy), A(zz):
  3.010279e-05  9.091089e-04 -9.392117e-04                  2.006853e-05  6.060726e-04 -6.261411e-04

Alignment Tensor Irreducible Representation (A0, A1R, A1I, A2R, A2I):
  1.013147e-03 -1.755171e-04  5.132035e-04 -7.859677e-04 -8.359289e-05

Tensor Properties:
A(axial)       =  -9.392117e-04         # alignment tensor axial component   = 3/2*A(zz) = S(zz)
A(rhombic)     =  -5.860041e-04         # alignment tensor rhombic component = A(xx) - A(yy) = 2/3*(S(xx) - S(yy))
A(rhombicity) =   6.239318e-01         # alignment tensor rhombicity        = A(rhombic) / A(axial)
A(asymmetry)   =   9.358978e-01         # alignment tensor asymmetry         = (A(xx) - A(yy))/A(zz) = (S(xx) - S(yy))/S(zz)
GDO            =   1.067553e-03         # generalized degree of order        = sqrt(3/2)*|A(xx),A(yy),A(zz)| = sqrt(2/3)*|S(xx),S(yy),S(zz)|

Results for Multi-Parameter SVD Fit of Calculated and Experimental Data:
           D(calc)[HZ]      +/- Error    D(exp)[HZ]      +/- Error  Rel. Weights           D(exp)-D(calc)        Normalized Weights    Atom Labels
D[01] =      -0.090675              -      0.100000      0.500000      1.000000     r[01] =       0.190675     w[01] =        0.090909 *) C1-H1
D[02] =      15.219659              -     15.350000      0.500000      1.000000     r[02] =       0.130341     w[02] =        0.090909 *) C2-H2
D[03] =      -7.629522              -     -7.650000      0.500000      1.000000     r[03] =      -0.020478     w[03] =        0.090909 *) C3-H3
D[04] =       5.920608              -      5.850000      0.500000      1.000000     r[04] =      -0.070608     w[04] =        0.090909 *) C4-H4s
D[05] =       3.046828              -      3.150000      0.500000      1.000000     r[05] =       0.103172     w[05] =        0.090909 *) C4-H4a
D[06] =       0.977797              -      1.100000      0.500000      1.000000     r[06] =       0.122203     w[06] =        0.090909 *) C5-H5
D[07] =     -11.007903              -    -10.850000      0.500000      1.000000     r[07] =       0.157903     w[07] =        0.090909 *) C7-H7s
D[08] =      18.064773              -     18.100000      0.500000      1.000000     r[08] =       0.035227     w[08] =        0.090909 *) C7-H7a
D[09] =       0.196600              -     -0.220000      0.500000      1.000000     r[09] =      -0.416600     w[09] =        0.090909    C6-C8
D[10] =      -0.423035              -      0.120000      0.500000      1.000000     r[10] =       0.543035     w[10] =        0.090909    C6-C9
D[11] =      -1.209479              -     -0.610000      0.500000      1.000000     r[11] =       0.599479     w[11] =        0.090909    C2-C10

Results for Multi-Parameter Fit of Calculated and Experimental Data:
rank   =              5                 # rank of cosine matrix (check input if rank < 5)
cond   =   3.855250e+00                 # condition number of cosine matrix (check input and singular values if very large)
chisq =       0.085302                 # weighted total sum of squared residuals
aic    =      13.753283                 # information criterion (AIC) for 5 degrees of freedom
rmsd   =       0.292065                 # unweighted root mean square deviation
qfac   =       0.034567                 # weighted Q-Factor as defined by Cornilescu
r^2    =       0.998716                 # coefficient of determination r^2 = 1 - chi^2 / (weighted sum of squares)
```

Table S1c. RDC data used for **1** (AM #3). The experimental data is listed as D_{exp}, and the RDCs back-calculated from the structure model are labeled D_{calc}; all values are given in [Hz]. In addition, the characteristics of the alignment tensor are listed.

```
SVD Best-Fit Saupe Vector S(zz), S(xx-yy), S(xy), S(xz), S(yz):
 -9.042809e-04  5.521761e-04  7.418978e-04 -8.273308e-04 -4.517361e-04
Saupe Tensor (S):                                      Alignment Tensor (A):
  7.282285e-04  7.418978e-04 -8.273308e-04               4.854857e-04  4.945985e-04 -5.515539e-04
  7.418978e-04  1.760524e-04 -4.517361e-04               4.945985e-04  1.173682e-04 -3.011574e-04
 -8.273308e-04 -4.517361e-04 -9.042809e-04              -5.515539e-04 -3.011574e-04 -6.028539e-04
Trace of Saupe Tensor:        0.000000e+00             Trace of Alignment Tensor:    0.000000e+00
Eigenvectors of Saupe Tensor (S):                      Eigenvectors of Alignment Tensor (A):
 -5.241167e-01 -3.439297e-01 -7.791110e-01              -5.241167e-01 -3.439297e-01 -7.791110e-01
  8.469968e-01 -1.150401e-01 -5.190011e-01               8.469968e-01 -1.150401e-01 -5.190011e-01
  8.887085e-02 -9.319217e-01  3.516019e-01               8.887085e-02 -9.319217e-01  3.516019e-01
Eigenvalues of Saupe Tensor S(xx), S(yy), S(zz):       Eigenvalues of Alignment Tensor A(xx), A(yy), A(zz):
 -3.304279e-04 -1.265375e-03  1.595803e-03              -2.202853e-04 -8.435833e-04  1.063869e-03

Alignment Tensor Irreducible Representation (A0, A1R, A1I, A2R, A2I):
 -1.433584e-03 -1.070911e-03 -5.847351e-04  3.573732e-04  9.603253e-04

Tensor Properties:
A(axial)      =   1.595803e-03         # alignment tensor axial component   = 3/2*A(zz) = S(zz)
A(rhombic)    =   6.232980e-04         # alignment tensor rhombic component = A(xx) - A(yy) = 2/3*(S(xx) - S(yy))
A(rhombicity) =   3.905859e-01         # alignment tensor rhombicity        = A(rhombic) / A(axial)
A(asymmetry)  =   5.858788e-01         # alignment tensor asymmetry         = (A(xx) - A(yy))/A(zz) = (S(xx) - S(yy))/S(zz)
GDO           =   1.684625e-03         # generalized degree of order        = sqrt(3/2)*|A(xx),A(yy),A(zz)| = sqrt(2/3)*|S(xx),S(yy),S(zz)|

Results for Multi-Parameter SVD Fit of Calculated and Experimental Data:
           D(calc)[HZ]     +/- Error    D(exp)[HZ]     +/- Error  Rel. Weights            D(exp)-D(calc)        Normalized Weights    Atom Labels
D[01] =       3.616290             -       3.500000      0.500000      1.000000    r[01] =      -0.116290     w[01] =       0.090909 *) C1-H1
D[02] =       6.641143             -       6.600000      0.500000      1.000000    r[02] =      -0.041143     w[02] =       0.090909 *) C2-H2
D[03] =      22.325292             -      22.350000      0.500000      1.000000    r[03] =       0.024708     w[03] =       0.090909 *) C3-H3
D[04] =      23.858659             -      23.800000      0.500000      1.000000    r[04] =      -0.058659     w[04] =       0.090909 *) C4-H4s
D[05] =     -11.061818             -     -11.250000      0.500000      1.000000    r[05] =      -0.188182     w[05] =       0.090909 *) C4-H4a
D[06] =       0.533412             -       0.400000      0.500000      1.000000    r[06] =      -0.133412     w[06] =       0.090909 *) C5-H5
D[07] =      -0.042118             -      -0.150000      0.500000      1.000000    r[07] =      -0.107882     w[07] =       0.090909 *) C7-H7s
D[08] =     -22.270348             -     -22.250000      0.500000      1.000000    r[08] =       0.020348     w[08] =       0.090909 *) C7-H7a
D[09] =      -2.049948             -      -2.250000      0.500000      1.000000    r[09] =      -0.200052     w[09] =       0.090909    C6-C8
D[10] =       2.107868             -       1.990000      0.500000      1.000000    r[10] =      -0.117868     w[10] =       0.090909    C6-C9
D[11] =       0.759433             -       0.520000      0.500000      1.000000    r[11] =      -0.239433     w[11] =       0.090909    C2-C10

Results for Multi-Parameter Fit of Calculated and Experimental Data:
rank  =              5                 # rank of cosine matrix (check input if rank < 5)
cond  =   3.855250e+00                 # condition number of cosine matrix (check input and singular values if very large)
chisq =       0.017798                 # weighted total sum of squared residuals
aic   =      10.783093                 # information criterion (AIC) for 5 degrees of freedom
rmsd  =       0.133408                 # unweighted root mean square deviation
qfac  =       0.010568                 # weighted Q-Factor as defined by Cornilescu
r^2   =       0.999885                 # coefficient of determination r^2 = 1 - chi^2 / (weighted sum of squares)
```

Table S1d. RDC data used for **1** (AM #4). The experimental data is listed as D_{exp}, and the RDCs back-calculated from the structure model are labeled D_{calc}; all values are given in [Hz]. In addition, the characteristics of the alignment tensor are listed.

```
SVD Best-Fit Saupe Vector S(zz), S(xx-yy), S(xy), S(xz), S(yz):
  1.477786e-04  2.148393e-04  1.501965e-05 -9.161081e-05 -1.828448e-04
Saupe Tensor (S):                                      Alignment Tensor (A):
  3.353037e-05  1.501965e-05 -9.161081e-05               2.235358e-05  1.001310e-05 -6.107387e-05
  1.501965e-05 -1.813089e-04 -1.828448e-04               1.001310e-05 -1.208726e-04 -1.218965e-04
 -9.161081e-05 -1.828448e-04  1.477786e-04              -6.107387e-05 -1.218965e-04  9.851904e-05
Trace of Saupe Tensor:        0.000000e+00             Trace of Alignment Tensor:    0.000000e+00
Eigenvectors of Saupe Tensor (S):                      Eigenvectors of Alignment Tensor (A):
  9.276701e-01  3.640036e-01  8.324413e-02               9.276701e-01  3.640036e-01  8.324413e-02
 -2.239662e-01  3.640331e-01  9.040570e-01              -2.239662e-01  3.640331e-01  9.040570e-01
  2.987763e-01 -8.573105e-01  4.192271e-01               2.987763e-01 -8.573105e-01  4.192271e-01
Eigenvalues of Saupe Tensor S(xx), S(yy), S(zz):       Eigenvalues of Alignment Tensor A(xx), A(yy), A(zz):
  3.989396e-07  2.643154e-04 -2.647143e-04               2.659597e-07  1.762102e-04 -1.764762e-04

Alignment Tensor Irreducible Representation (A0, A1R, A1I, A2R, A2I):
  2.342779e-04 -1.185826e-04 -2.366775e-04  1.390458e-04  1.944170e-05

Tensor Properties:
A(axial)      =  -2.647143e-04         # alignment tensor axial component   = 3/2*A(zz) = S(zz)
A(rhombic)    =  -1.759443e-04         # alignment tensor rhombic component = A(xx) - A(yy) = 2/3*(S(xx) - S(yy))
A(rhombicity) =   6.646573e-01         # alignment tensor rhombicity        = A(rhombic) / A(axial)
A(asymmetry)  =   9.969859e-01         # alignment tensor asymmetry         = (A(xx) - A(yy))/A(zz) = (S(xx) - S(yy))/S(zz)
GDO           =   3.054357e-04         # generalized degree of order        = sqrt(3/2)*|A(xx),A(yy),A(zz)| = sqrt(2/3)*|S(xx),S(yy),S(zz)|

Results for Multi-Parameter SVD Fit of Calculated and Experimental Data:
           D(calc)[HZ]     +/- Error    D(exp)[HZ]     +/- Error  Rel. Weights            D(exp)-D(calc)        Normalized Weights    Atom Labels
D[01] =      -2.493028             -      -2.100000      0.500000      1.000000    r[01] =       0.393028     w[01] =       0.090909 *) C1-H1
D[02] =       0.679028             -       0.500000      0.500000      1.000000    r[02] =      -0.179028     w[02] =       0.090909 *) C2-H2
D[03] =       2.556608             -       2.600000      0.500000      1.000000    r[03] =       0.043392     w[03] =       0.090909 *) C3-H3
D[04] =      -1.415932             -      -1.100000      0.500000      1.000000    r[04] =       0.315932     w[04] =       0.090909 *) C4-H4s
D[05] =       3.756801             -       4.300000      0.500000      1.000000    r[05] =       0.543199     w[05] =       0.090909 *) C4-H4a
D[06] =       1.347418             -       1.850000      0.500000      1.000000    r[06] =       0.502582     w[06] =       0.090909 *) C5-H5
D[07] =      -4.595028             -      -4.250000      0.500000      1.000000    r[07] =       0.345028     w[07] =       0.090909 *) C7-H7s
D[08] =       0.413325             -       0.600000      0.500000      1.000000    r[08] =       0.186675     w[08] =       0.090909 *) C7-H7a
D[09] =      -0.454992             -      -0.630000      0.500000      1.000000    r[09] =      -0.175008     w[09] =       0.090909    C6-C8
D[10] =       0.066216             -       0.350000      0.500000      1.000000    r[10] =       0.283784     w[10] =       0.090909    C6-C9
D[11] =       0.454522             -       0.740000      0.500000      1.000000    r[11] =       0.285478     w[11] =       0.090909    C2-C10

Results for Multi-Parameter Fit of Calculated and Experimental Data:
rank  =              5                 # rank of cosine matrix (check input if rank < 5)
cond  =   3.855250e+00                 # condition number of cosine matrix (check input and singular values if very large)
chisq =       0.107493                 # weighted total sum of squared residuals
aic   =      14.729686                 # information criterion (AIC) for 5 degrees of freedom
rmsd  =       0.327861                 # unweighted root mean square deviation
qfac  =       0.147931                 # weighted Q-Factor as defined by Cornilescu
r^2   =       0.977811                 # coefficient of determination r^2 = 1 - chi^2 / (weighted sum of squares)
```

RDC Data:

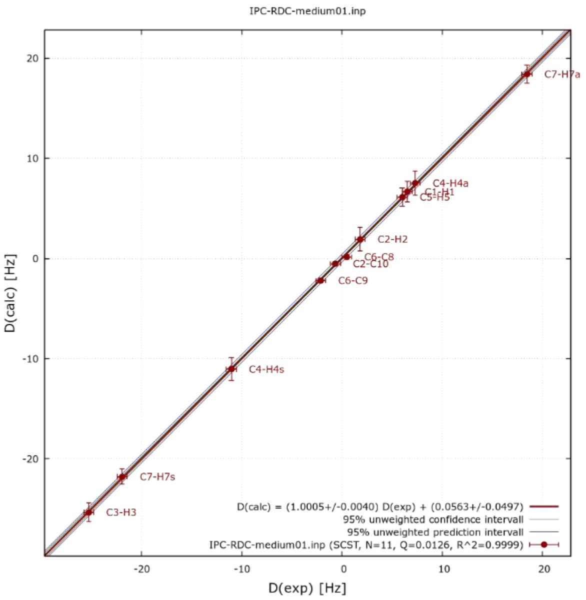

(AM #1)

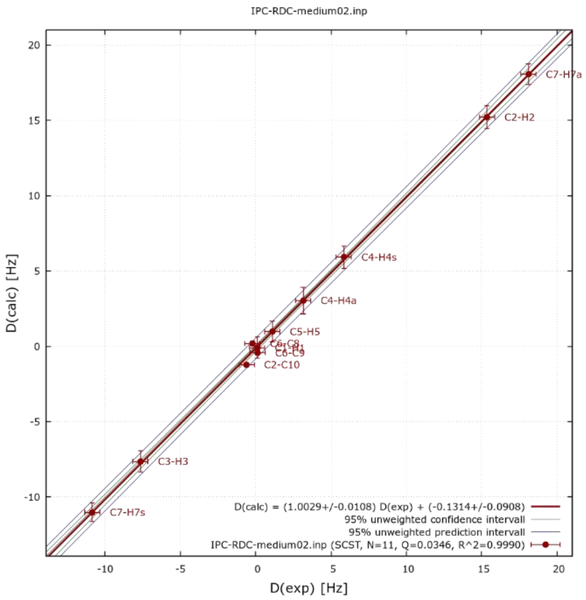

(AM #2)

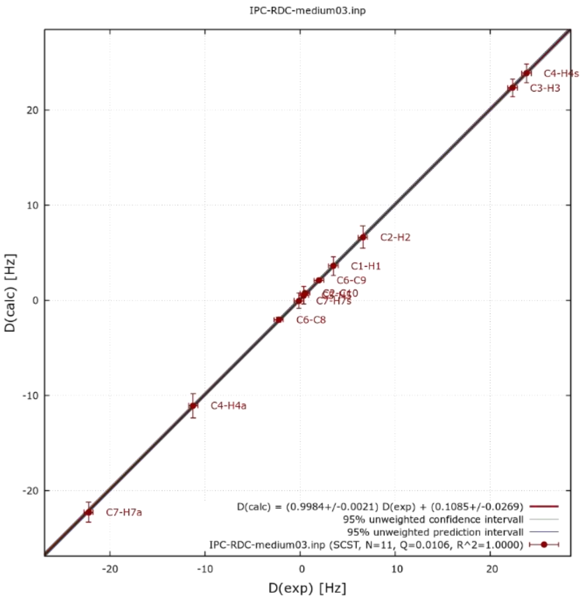

(AM #3)

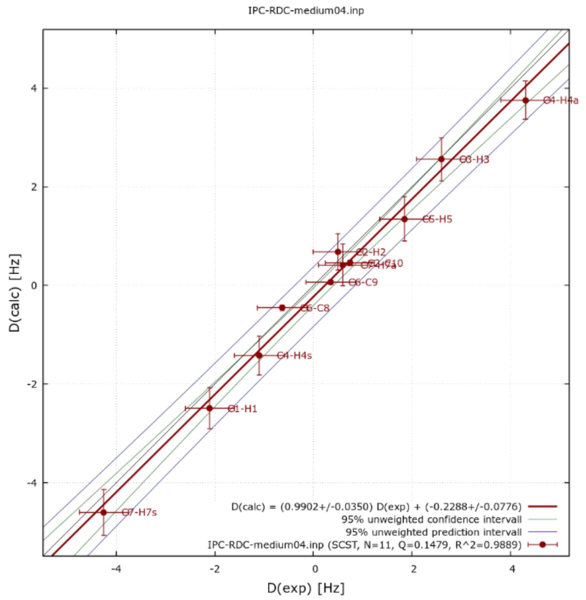

(AM #4)

Alignment Tensors:

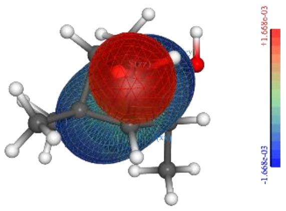

(AM #1)

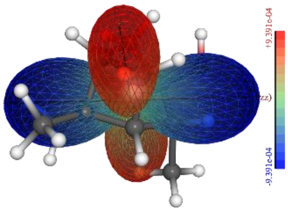

(AM #2)

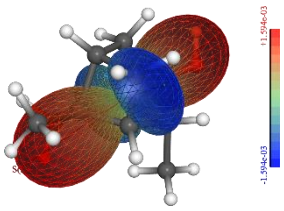

(AM #3)

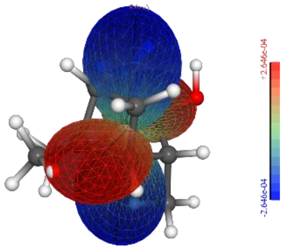

(AM #4)

Figure S1. Plot of back-calculated vs. experimental RDC data (alignment media #1-#4) used for the configurational analysis of IPC (**1**).

RDC and NOE Data for Plakilactone H (2)

The following Tables S2a-b list the experimental and back-calculated RDC and NOE data used for the configurational analysis of plakilactone (**2**). The Tables were generated directly from the output files for the *ConArch*$^{+}$*/DG* best-fit (minimum pseudo energy) structure obtained from fc-rDG/DDD configurational and conformational analysis using an experimental NOE data set and a single alignment medium RDC data set ($K_{NOE} = 50$, $K_{RDC} = 0.5$ Hz^{-2}).

Table S2a. NOE data used for **2**.

```
Results for Calculated and Experimental NOEs:
           d(averaged) d(exp)-d(calc)  (Percent) d(lower) d(expmean) d(upper) (+/-Range)       Weights    NOE Contact
NOE[01] =     2.677924      0.072076   (  -2.6%)  2.47500   2.75000   3.02500  (  10.0%)      1.000000    H16A|H16B|H16C || H15B
NOE[02] =     3.065476      0.254524   (  -7.7%)  2.98800   3.32000   3.65200  (  10.0%)      1.000000    H16A|H16B|H16C || H8
NOE[03] =     3.215662     -0.075662   (   2.4%)  2.82600   3.14000   3.45400  (  10.0%)      1.000000    H3 || H11A|H11B
NOE[04] =     2.780041      0.119959   (  -4.1%)  2.61000   2.90000   3.19000  (  10.0%)      1.000000    H3 || H5B
NOE[05] =     3.644640     -0.794640   (  27.9%)  2.56500   2.85000   3.13500  (  10.0%)      1.000000    H3 || H13A
NOE[06] =     3.243576     -0.043576   (   1.4%)  2.88000   3.20000   3.52000  (  10.0%)      1.000000    H3 || H12A|H12B|H12C
NOE[07] =     2.879551      0.230449   (  -7.4%)  2.79900   3.11000   3.42100  (  10.0%)      1.000000    H3 || H14A|H14B|H14C
NOE[08] =     3.287369     -0.067369   (   2.1%)  2.89800   3.22000   3.54200  (  10.0%)      1.000000    H3 || H6
NOE[09] =     2.709214     -0.029214   (   1.1%)  2.41200   2.68000   2.94800  (  10.0%)      1.000000    H8 || H9A|H9B
NOE[10] =     3.203075      0.006925   (  -0.2%)  2.88900   3.21000   3.53100  (  10.0%)      1.000000    H8 || H10A|H10B|H10C
NOE[11] =     2.395295     -0.075295   (   3.2%)  2.08800   2.32000   2.55200  (  10.0%)      1.000000    H8 || H6
NOE[12] =     2.599658      0.040342   (  -1.5%)  2.37600   2.64000   2.90400  (  10.0%)      1.000000    H7 || H5B
NOE[13] =     2.749808     -0.099808   (   3.8%)  2.38500   2.65000   2.91500  (  10.0%)      1.000000    H7 || H9A|H9B
NOE[14] =     2.506824      0.223176   (  -8.2%)  2.45700   2.73000   3.00300  (  10.0%)      1.000000    H7 || H15A
NOE[15] =     3.491043      0.048957   (  -1.4%)  3.18600   3.54000   3.89400  (  10.0%)      1.000000    H7 || H10A|H10B|H10C
NOE[16] =     3.793415     -0.353415   (  10.3%)  3.09600   3.44000   3.78400  (  10.0%)      1.000000    H7 || H16A|H16B|H16C
NOE[17] =     3.047829     -0.107829   (   3.7%)  2.64600   2.94000   3.23400  (  10.0%)      1.000000    H7 || H6
NOE[18] =     2.534669      0.135331   (  -5.1%)  2.40300   2.67000   2.93700  (  10.0%)      1.000000    H5B || H13A
NOE[19] =     2.885903      0.344097   ( -10.7%)  2.90700   3.23000   3.55300  (  10.0%)      1.000000    H5B || H6
NOE[20] =     3.129243     -0.089243   (   2.9%)  2.73600   3.04000   3.34400  (  10.0%)      1.000000    H5B || H14A|H14B|H14C
NOE[21] =     2.720051      0.249949   (  -8.4%)  2.67300   2.97000   3.26700  (  10.0%)      1.000000    H15B || H6
NOE[22] =     4.020436     -0.580436   (  16.9%)  3.09600   3.44000   3.78400  (  10.0%)      1.000000    H15A || H8
NOE[23] =     2.650959      0.089041   (  -3.2%)  2.46600   2.74000   3.01400  (  10.0%)      1.000000    H9A|H9B || H10A|H10B|H10C
NOE[24] =     2.679017      0.340983   ( -11.3%)  2.71800   3.02000   3.32200  (  10.0%)      1.000000    H15A || H16A|H16B|H16C
NOE[25] =     2.639488      0.120512   (  -4.4%)  2.48400   2.76000   3.03600  (  10.0%)      1.000000    H12A|H12B|H12C || H11A|H11B
----------
*) used r^-6 for averaging NOEs.
```

Table S2b. RDC data used for **2** (AM #1). The experimental data is listed as D_{exp}, and the RDCs back-calculated from the structure model are labeled D_{calc}; all values are given in [Hz]. In addition, the characteristics of the alignment tensor are listed.

```
SVD Best-Fit Saupe Vector S(zz), S(xx-yy), S(xy), S(xz), S(yz):
 -1.444571e-03 -8.857526e-04 -6.555844e-04  2.522915e-04 -1.023944e-03
Saupe Tensor (S):                                     Alignment Tensor (A):
  2.794093e-04 -6.555844e-04  2.522915e-04              1.862729e-04 -4.370563e-04  1.681944e-04
 -6.555844e-04  1.165162e-03 -1.023944e-03             -4.370563e-04  7.767746e-04 -6.826294e-04
  2.522915e-04 -1.023944e-03 -1.444571e-03              1.681944e-04 -6.826294e-04 -9.630475e-04
Trace of Saupe Tensor:        0.000000e+00            Trace of Alignment Tensor:    0.000000e+00
Eigenvectors of Saupe Tensor (S):                     Eigenvectors of Alignment Tensor (A):
  9.114426e-01  1.262561e-02 -4.112335e-01              9.114426e-01  1.262561e-02 -4.112335e-01
  3.928042e-01 -3.240197e-01  8.606486e-01              3.928042e-01 -3.240197e-01  8.606486e-01
 -1.223815e-01 -9.459661e-01 -3.002848e-01             -1.223815e-01 -9.459661e-01 -3.002848e-01
Eigenvalues of Saupe Tensor S(xx), S(yy), S(zz):      Eigenvalues of Alignment Tensor A(xx), A(yy), A(zz):
 -3.700353e-05 -1.798668e-03  1.835671e-03             -2.466902e-05 -1.199112e-03  1.223781e-03

Alignment Tensor Irreducible Representation (A0, A1R, A1I, A2R, A2I):
 -2.290123e-03  3.265705e-04 -1.325411e-03 -5.732668e-04 -8.485997e-04

Tensor Properties:
A(axial)      =   1.835671e-03         # alignment tensor axial component   = 3/2*A(zz) = S(zz)
A(rhombic)    =   1.174443e-03         # alignment tensor rhombic component = A(xx) - A(yy) = 2/3*(S(xx) - S(yy))
A(rhombicity) =   6.397893e-01         # alignment tensor rhombicity        = A(rhombic) / A(axial)
A(asymmetry)  =   9.596839e-01         # alignment tensor asymmetry         = (A(xx) - A(yy))/A(zz) = (S(xx) - S(yy))/S(zz)
GDO           =   2.098613e-03         # generalized degree of order        = sqrt(3/2)*|A(xx),A(yy),A(zz)| = sqrt(2/3)*|S(xx),S(yy),S(zz)|

Results for Multi-Parameter SVD Fit of Calculated and Experimental Data:
          D(calc)[HZ]     +/- Error    D(exp)[HZ]     +/- Error  Rel. Weights            D(exp)-D(calc)       Normalized Weights    Atom Labels
D[01] =     22.597286             -     22.598000      0.500000      1.000000    r[01] =        0.000714    w[01] =        0.076923 *) C3-H3
D[02] =    -18.292293             -    -18.294000      0.500000      1.000000    r[02] =       -0.001707    w[02] =        0.076923 *) C6-H6
D[03] =    -14.544692             -    -14.552000      0.500000      1.000000    r[03] =       -0.007308    w[03] =        0.076923 *) C7-H7
D[04] =     20.053458             -     20.057000      0.500000      1.000000    r[04] =        0.003542    w[04] =        0.076923 *) C8-H8
D[05] =     -0.252508             -     -0.292000      0.500000      1.000000    r[05] =       -0.039492    w[05] =        0.076923 *) C5-H5A+C5-H5B
D[06] =      8.141170             -      8.169000      0.500000      1.000000    r[06] =        0.027830    w[06] =        0.076923 *) C9-H9A+C9-H9B
D[07] =     26.042241             -     26.028000      0.500000      1.000000    r[07] =       -0.014241    w[07] =        0.076923 *) C11-H11A+C11-H11B
D[08] =    -28.773159             -    -28.760000      0.500000      1.000000    r[08] =        0.013159    w[08] =        0.076923 *) C13-H13A+C13-H13B
D[09] =    -17.197990             -    -17.206000      0.500000      1.000000    r[09] =       -0.008010    w[09] =        0.076923 *) C15-H15A+C15-H15B
D[10] =      1.648206             -      1.632000      0.500000      1.000000    r[10] =       -0.016206    w[10] =        0.076923 *) C10-H10A|H10B|H10C
D[11] =      0.780395             -      0.816000      0.500000      1.000000    r[11] =        0.035605    w[11] =        0.076923 *) C12-H12A|H12B|H12C
D[12] =    -10.545312             -    -10.614000      0.500000      1.000000    r[12] =       -0.068688    w[12] =        0.076923 *) C14-H14A|H14B|H14C
D[13] =     -5.315158             -     -5.207000      0.500000      1.000000    r[13] =        0.108158    w[13] =        0.076923 *) C16-H16A|H16B|H16C

Results for Multi-Parameter Fit of Calculated and Experimental Data:
rank  =             5                  # rank of cosine matrix (check input if rank < 5)
cond  =   3.749357e+00                 # condition number of cosine matrix (check input and singular values if very large)
chisq =      0.001599                  # weighted total sum of squared residuals
aic   =     10.083161                  # information criterion (AIC) for 5 degrees of freedom
rmsd  =      0.039991                  # unweighted root mean square deviation
qfac  =      0.002448                  # weighted Q-Factor as defined by Cornilescu
r^2   =      0.999994                  # coefficient of determination r^2 = 1 - chi^2 / (weighted sum of squares)
```

RDC and NOE Data for Vincristine (3)

The following Tables S3a-d list the back-calculated RDC and NOE data used for the configurational analysis of vincristine (**3**). The Tables were generated directly from the output files for the *ConArch*$^{+}$*/DG* best-fit (minimum pseudo energy) structure obtained from fc-rDG/DDD configurational and conformational analysis using a NOE data set and three alignment media RDC data sets ($K_{NOE} = 60$, $K_{RDC} = 0.5$ Hz^{-2}).

Table S3a. NOE data used for **3**.

```
Results for Calculated and Experimental NOEs:
           d(averaged) d(exp)-d(calc)  (Percent) d(lower) d(expmean) d(upper) (+/-Range)      Weights    NOE Contact
NOE[01] =     2.184410      0.020590   (  -0.9%) 1.98450  2.20500   2.42550  (  10.0%)      1.000000   H30 || H35
NOE[02] =     2.511022     -0.053022   (   2.2%) 2.21220  2.45800   2.70380  (  10.0%)      1.000000   H27 || H39
NOE[03] =     2.786951     -0.114951   (   4.3%) 2.40480  2.67200   2.93920  (  10.0%)      1.000000   H1 || H27
NOE[04] =     2.692357     -0.008357   (   0.3%) 2.41560  2.68400   2.95240  (  10.0%)      1.000000   H15 || H27
NOE[05] =     2.283756     -0.107756   (   5.0%) 1.95840  2.17600   2.39360  (  10.0%)      1.000000   H10A|H10B || H12A|H12B
NOE[06] =     2.807607     -0.024607   (   0.9%) 2.50470  2.78300   3.06130  (  10.0%)      1.000000   H10A|H10B || H16A|H16B
NOE[07] =     2.722732     -0.017732   (   0.7%) 2.43450  2.70500   2.97550  (  10.0%)      1.000000   H12A|H12B || H18A|H18B
NOE[08] =     3.148979     -0.048979   (   1.6%) 2.79000  3.10000   3.41000  (  10.0%)      1.000000   H14A|H14B || H16A|H16B
NOE[09] =     2.932560     -0.006560   (   0.2%) 2.63340  2.92600   3.21860  (  10.0%)      1.000000   H14A|H14B || H18A|H18B
NOE[10] =     2.586219      0.011781   (  -0.5%) 2.33820  2.59800   2.85780  (  10.0%)      1.000000   H14A|H14B || H27
NOE[11] =     3.302755     -0.010755   (   0.3%) 2.96280  3.29200   3.62120  (  10.0%)      1.000000   H16A|H16B || H18A|H18B
NOE[12] =     2.793354      0.100646   (  -3.5%) 2.60460  2.89400   3.18340  (  10.0%)      1.000000   H27 || H43A|H43B
NOE[13] =     3.424424     -0.120424   (   3.6%) 2.97360  3.30400   3.63440  (  10.0%)      1.000000   H27 || H58A|H58B
NOE[14] =     2.541976      0.020024   (  -0.8%) 2.30580  2.56200   2.81820  (  10.0%)      1.000000   H37 || H43A|H43B
NOE[15] =     3.314125     -0.027125   (   0.8%) 2.95830  3.28700   3.61570  (  10.0%)      1.000000   H37 || H44A|H44B
NOE[16] =     3.451335      0.011665   (  -0.3%) 3.11670  3.46300   3.80930  (  10.0%)      1.000000   H39 || H41
NOE[17] =     3.182756     -0.042756   (   1.4%) 2.82600  3.14000   3.45400  (  10.0%)      1.000000   H39 || H43A|H43B
NOE[18] =     3.397465     -0.067465   (   2.0%) 2.99700  3.33000   3.66300  (  10.0%)      1.000000   H39 || H44A|H44B
NOE[19] =     3.072722     -0.058722   (   1.9%) 2.71260  3.01400   3.31540  (  10.0%)      1.000000   H39 || H46A|H46B
NOE[20] =     2.614940      0.038060   (  -1.4%) 2.38770  2.65300   2.91830  (  10.0%)      1.000000   H39 || H58A|H58B
NOE[21] =     2.372189     -0.011189   (   0.5%) 2.12490  2.36100   2.59710  (  10.0%)      1.000000   H41 || H58A|H58B
NOE[22] =     2.807896      0.021104   (  -0.7%) 2.54610  2.82900   3.11190  (  10.0%)      1.000000   H44A|H44B || H46A|H46B
NOE[23] =     2.940008     -0.076008   (   2.7%) 2.57760  2.86400   3.15040  (  10.0%)      1.000000   H48 || H58A|H58B
----------
*) used r^-6 for averaging NOEs.
```

Table S3b. RDC data used for **3** (AM #1). The experimental data is listed as D_{exp}, and the RDCs back-calculated from the structure model are labeled D_{calc}; all values are given in [Hz]. In addition, the characteristics of the alignment tensor are listed.

```
SVD Best-Fit Saupe Vector S(zz), S(xx-yy), S(xy), S(xz), S(yz):
 -1.169867e-03 -6.993955e-04  6.441956e-04  3.279541e-04 -1.651919e-04
Saupe Tensor (S):                                       Alignment Tensor (A):
  2.352357e-04  6.441956e-04  3.279541e-04                1.568238e-04  4.294637e-04  2.186360e-04
  6.441956e-04  9.346311e-04 -1.651919e-04                4.294637e-04  6.230874e-04 -1.101279e-04
  3.279541e-04 -1.651919e-04 -1.169867e-03                2.186360e-04 -1.101279e-04 -7.799112e-04
Trace of Saupe Tensor:        0.000000e+00              Trace of Alignment Tensor:    0.000000e+00
Eigenvectors of Saupe Tensor (S):                       Eigenvectors of Alignment Tensor (A):
 -8.151852e-01  2.677394e-01 -5.136037e-01               -8.151852e-01  2.677394e-01 -5.136037e-01
  4.918248e-01 -1.483657e-01 -8.579604e-01                4.918248e-01 -1.483657e-01 -8.579604e-01
 -3.059110e-01 -9.519996e-01 -1.073535e-02               -3.059110e-01 -9.519996e-01 -1.073535e-02
Eigenvalues of Saupe Tensor S(xx), S(yy), S(zz):        Eigenvalues of Alignment Tensor A(xx), A(yy), A(zz):
 -3.035622e-05 -1.287845e-03  1.318201e-03               -2.023748e-05 -8.585632e-04  8.788007e-04

Alignment Tensor Irreducible Representation (A0, A1R, A1I, A2R, A2I):
 -1.854626e-03  4.245094e-04 -2.138273e-04 -4.526548e-04  8.338579e-04

Tensor Properties:
A(axial)      =   1.318201e-03         # alignment tensor axial component   = 3/2*A(zz) = S(zz)
A(rhombic)    =   8.383257e-04         # alignment tensor rhombic component = A(xx) - A(yy) = 2/3*(S(xx) - S(yy))
A(rhombicity) =   6.359620e-01         # alignment tensor rhombicity        = A(rhombic) / A(axial)
A(asymmetry)  =   9.539430e-01         # alignment tensor asymmetry         = (A(xx) - A(yy))/A(zz) = (S(xx) - S(yy))/S(zz)
GDO           =   1.504907e-03         # generalized degree of order        = sqrt(3/2)*|A(xx),A(yy),A(zz)| = sqrt(2/3)*|S(xx),S(yy),S(zz)|

Results for Multi-Parameter SVD Fit of Calculated and Experimental Data:
           D(calc)[HZ]     +/- Error    D(exp)[HZ]     +/- Error  Rel. Weights           D(exp)-D(calc)       Normalized Weights    Atom Labels
D[01] =      18.621430          -       18.600000      0.500000      1.000000    r[01] =      -0.021430    w[01] =        0.041667 *) C5-H5
D[02] =       4.520640          -        4.470000      0.500000      1.000000    r[02] =      -0.050640    w[02] =        0.041667 *) C6-H6
D[03] =      -0.131160          -       -0.130000      0.500000      1.000000    r[03] =       0.001160    w[03] =        0.041667 *) C7-H7
D[04] =      19.935834          -       19.980000      0.500000      1.000000    r[04] =       0.044166    w[04] =        0.041667 *) C8-H8
D[05] =      -4.790050          -       -4.790000      0.500000      1.000000    r[05] =       0.000050    w[05] =        0.041667 *) C10-H10A+C10-H10B
D[06] =      -8.643737          -       -8.620000      0.500000      1.000000    r[06] =       0.023737    w[06] =        0.041667 *) C11-H11A+C11-H11B
D[07] =      29.212940          -       29.220000      0.500000      1.000000    r[07] =       0.007060    w[07] =        0.041667 *) C12-H12A+C12-H12B
D[08] =     -13.513054          -      -13.510000      0.500000      1.000000    r[08] =       0.003054    w[08] =        0.041667 *) C14-H14A+C14-H14B
D[09] =      -8.527241          -       -8.570000      0.500000      1.000000    r[09] =      -0.042759    w[09] =        0.041667 *) C15-H15
D[10] =      -3.058739          -       -3.060000      0.500000      1.000000    r[10] =      -0.001261    w[10] =        0.041667 *) C16-H16A+C16-H16B
D[11] =     -17.530475          -      -17.520000      0.500000      1.000000    r[11] =       0.010475    w[11] =        0.041667 *) C18-H18A+C18-H18B
D[12] =       0.944355          -        0.940000      0.500000      1.000000    r[12] =      -0.004355    w[12] =        0.041667 *) C24-H24A+C24-H24B
D[13] =      14.441520          -       14.520000      0.500000      1.000000    r[13] =       0.078480    w[13] =        0.041667 *) C27-H27
D[14] =      16.997674          -       17.020000      0.500000      1.000000    r[14] =       0.022326    w[14] =        0.041667 *) C30-H30
D[15] =       8.047815          -        7.910000      0.500000      1.000000    r[15] =      -0.137815    w[15] =        0.041667 *) C35-H35
D[16] =     -16.641372          -      -16.610000      0.500000      1.000000    r[16] =       0.031372    w[16] =        0.041667 *) C37-H37
D[17] =     -24.928046          -      -24.870000      0.500000      1.000000    r[17] =       0.058046    w[17] =        0.041667 *) C39-H39
D[18] =      -5.063665          -       -5.030000      0.500000      1.000000    r[18] =       0.033665    w[18] =        0.041667 *) C41-H41
D[19] =     -30.786395          -      -30.830000      0.500000      1.000000    r[19] =      -0.043605    w[19] =        0.041667 *) C43-H43A+C43-H43B
D[20] =     -14.425929          -      -14.420000      0.500000      1.000000    r[20] =       0.005929    w[20] =        0.041667 *) C44-H44A+C44-H44B
D[21] =     -22.396563          -      -22.400000      0.500000      1.000000    r[21] =      -0.003437    w[21] =        0.041667 *) C46-H46A+C46-H46B
D[22] =      -0.971077          -       -0.880000      0.500000      1.000000    r[22] =       0.091077    w[22] =        0.041667 *) C47-H47
D[23] =      15.501308          -       15.490000      0.500000      1.000000    r[23] =      -0.011308    w[23] =        0.041667 *) C48-H48
D[24] =      22.247731          -       22.260000      0.500000      1.000000    r[24] =       0.012269    w[24] =        0.041667 *) C58-H58A+C58-H58B

Results for Multi-Parameter Fit of Calculated and Experimental Data:
rank  =              5                 # rank of cosine matrix (check input if rank < 5)
cond  =   3.284159e+00                 # condition number of cosine matrix (check input and singular values if very large)
chisq =       0.002051                 # weighted total sum of squared residuals
aic   =      10.196864                 # information criterion (AIC) for 5 degrees of freedom
rmsd  =       0.045284                 # unweighted root mean square deviation
qfac  =       0.002825                 # weighted Q-Factor as defined by Cornilescu
r^2   =       0.999992                 # coefficient of determination r^2 = 1 - chi^2 / (weighted sum of squares)
```

Table S3c. RDC data used for **3** (AM #2). The experimental data is listed as D_{exp}, and the RDCs back-calculated from the structure model are labeled D_{calc}; all values are given in [Hz]. In addition, the characteristics of the alignment tensor are listed.

```
SVD Best-Fit Saupe Vector S(zz), S(xx-yy), S(xy), S(xz), S(yz):
 -2.737647e-04  2.061993e-03  4.011437e-04 -5.874823e-04  4.205973e-04
Saupe Tensor (S):                                       Alignment Tensor (A):
  1.167879e-03  4.011437e-04 -5.874823e-04                7.785858e-04  2.674291e-04 -3.916549e-04
  4.011437e-04 -8.941141e-04  4.205973e-04                2.674291e-04 -5.960761e-04  2.803982e-04
 -5.874823e-04  4.205973e-04 -2.737647e-04               -3.916549e-04  2.803982e-04 -1.825098e-04
Trace of Saupe Tensor:        5.421011e-20              Trace of Alignment Tensor:    3.614007e-20
Eigenvectors of Saupe Tensor (S):                       Eigenvectors of Alignment Tensor (A):
  1.969020e-01  2.562995e-01  9.463298e-01                1.969020e-01  2.562995e-01  9.463298e-01
  5.505628e-01 -8.275704e-01  1.095801e-01                5.505628e-01 -8.275704e-01  1.095801e-01
  8.112399e-01  4.994375e-01 -3.040592e-01                8.112399e-01  4.994375e-01 -3.040592e-01
Eigenvalues of Saupe Tensor S(xx), S(yy), S(zz):        Eigenvalues of Alignment Tensor A(xx), A(yy), A(zz):
 -1.309108e-04 -1.272179e-03  1.403089e-03               -8.727386e-05 -8.481190e-04  9.353929e-04

Alignment Tensor Irreducible Representation (A0, A1R, A1I, A2R, A2I):
 -4.340076e-04 -7.604473e-04  5.444284e-04  1.334540e-03  5.192474e-04

Tensor Properties:
A(axial)      =   1.403089e-03        # alignment tensor axial component   = 3/2*A(zz) = S(zz)
A(rhombic)    =   7.608452e-04        # alignment tensor rhombic component = A(xx) - A(yy) = 2/3*(S(xx) - S(yy))
A(rhombicity) =   5.422642e-01        # alignment tensor rhombicity        = A(rhombic) / A(axial)
A(asymmetry)  =   8.133964e-01        # alignment tensor asymmetry         = (A(xx) - A(yy))/A(zz) = (S(xx) - S(yy))/S(zz)
GDO           =   1.550104e-03        # generalized degree of order        = sqrt(3/2)*|A(xx),A(yy),A(zz)| = sqrt(2/3)*|S(xx),S(yy),S(zz)|

Results for Multi-Parameter SVD Fit of Calculated and Experimental Data:
           D(calc)[HZ]     +/- Error    D(exp)[HZ]     +/- Error  Rel. Weights            D(exp)-D(calc)        Normalized Weights    Atom Labels
D[01] =     -25.302933           -     -25.150000     0.500000     1.000000    r[01] =       0.152933    w[01] =       0.041667 *) C5-H5
D[02] =      20.892224           -      20.860000     0.500000     1.000000    r[02] =      -0.032224    w[02] =       0.041667 *) C6-H6
D[03] =      10.229294           -      10.270000     0.500000     1.000000    r[03] =       0.040706    w[03] =       0.041667 *) C7-H7
D[04] =     -24.217254           -     -24.190000     0.500000     1.000000    r[04] =       0.027254    w[04] =       0.041667 *) C8-H8
D[05] =       0.501181           -       0.490000     0.500000     1.000000    r[05] =      -0.011181    w[05] =       0.041667 *) C10-H10A+C10-H10B
D[06] =       8.210967           -       8.170000     0.500000     1.000000    r[06] =      -0.040967    w[06] =       0.041667 *) C11-H11A+C11-H11B
D[07] =     -10.680727           -     -10.720000     0.500000     1.000000    r[07] =      -0.039273    w[07] =       0.041667 *) C12-H12A+C12-H12B
D[08] =      10.348056           -      10.350000     0.500000     1.000000    r[08] =       0.001944    w[08] =       0.041667 *) C14-H14A+C14-H14B
D[09] =     -27.622369           -     -27.710000     0.500000     1.000000    r[09] =      -0.087631    w[09] =       0.041667 *) C15-H15
D[10] =      17.719358           -      17.720000     0.500000     1.000000    r[10] =       0.000642    w[10] =       0.041667 *) C16-H16A+C16-H16B
D[11] =     -25.694746           -     -25.700000     0.500000     1.000000    r[11] =      -0.005254    w[11] =       0.041667 *) C18-H18A+C18-H18B
D[12] =      15.747272           -      15.750000     0.500000     1.000000    r[12] =       0.002728    w[12] =       0.041667 *) C24-H24A+C24-H24B
D[13] =      18.672036           -      18.780000     0.500000     1.000000    r[13] =       0.107964    w[13] =       0.041667 *) C27-H27
D[14] =      14.888563           -      14.740000     0.500000     1.000000    r[14] =      -0.148563    w[14] =       0.041667 *) C30-H30
D[15] =       6.306516           -       6.560000     0.500000     1.000000    r[15] =       0.253484    w[15] =       0.041667 *) C35-H35
D[16] =       8.677268           -       8.660000     0.500000     1.000000    r[16] =      -0.017268    w[16] =       0.041667 *) C37-H37
D[17] =       1.849034           -       2.000000     0.500000     1.000000    r[17] =       0.150966    w[17] =       0.041667 *) C39-H39
D[18] =      -4.614806           -      -4.670000     0.500000     1.000000    r[18] =      -0.055194    w[18] =       0.041667 *) C41-H41
D[19] =       3.434207           -       3.450000     0.500000     1.000000    r[19] =       0.015793    w[19] =       0.041667 *) C43-H43A+C43-H43B
D[20] =       6.783892           -       6.810000     0.500000     1.000000    r[20] =       0.026108    w[20] =       0.041667 *) C44-H44A+C44-H44B
D[21] =      -2.483554           -      -2.470000     0.500000     1.000000    r[21] =       0.013554    w[21] =       0.041667 *) C46-H46A+C46-H46B
D[22] =      -3.266621           -      -3.290000     0.500000     1.000000    r[22] =      -0.023379    w[22] =       0.041667 *) C47-H47
D[23] =     -13.530778           -     -13.540000     0.500000     1.000000    r[23] =      -0.009222    w[23] =       0.041667 *) C48-H48
D[24] =     -24.944268           -     -24.940000     0.500000     1.000000    r[24] =       0.004268    w[24] =       0.041667 *) C58-H58A+C58-H58B

Results for Multi-Parameter Fit of Calculated and Experimental Data:
rank  =              5                # rank of cosine matrix (check input if rank < 5)
cond  =   3.284159e+00                # condition number of cosine matrix (check input and singular values if very large)
chisq =       0.006824                # weighted total sum of squared residuals
aic   =      10.655087                # information criterion (AIC) for 5 degrees of freedom
rmsd  =       0.082606                # unweighted root mean square deviation
qfac  =       0.005392                # weighted Q-Factor as defined by Cornilescu
r^2   =       0.999971                # coefficient of determination r^2 = 1 - chi^2 / (weighted sum of squares)
```

Table S3d. RDC data used for **3** (AM #3). The experimental data is listed as D_{exp}, and the RDCs back-calculated from the structure model are labeled D_{calc}; all values are given in [Hz]. In addition, the characteristics of the alignment tensor are listed.

```
SVD Best-Fit Saupe Vector S(zz), S(xx-yy), S(xy), S(xz), S(yz):
 -1.711292e-05  3.416703e-04  4.723710e-04  3.350407e-04  7.770522e-04
Saupe Tensor (S):                                       Alignment Tensor (A):
  1.793916e-04  4.723710e-04  3.350407e-04                1.195944e-04  3.149140e-04  2.233605e-04
  4.723710e-04 -1.622787e-04  7.770522e-04                3.149140e-04 -1.081858e-04  5.180348e-04
  3.350407e-04  7.770522e-04 -1.711292e-05                2.233605e-04  5.180348e-04 -1.140861e-05
Trace of Saupe Tensor:       -1.355253e-20              Trace of Alignment Tensor:   -9.035018e-21
Eigenvectors of Saupe Tensor (S):                       Eigenvectors of Alignment Tensor (A):
 -8.268147e-01 -1.413454e-01 -5.444253e-01               -8.268147e-01 -1.413454e-01 -5.444253e-01
  2.578312e-01  7.649934e-01 -5.901764e-01                2.578312e-01  7.649934e-01 -5.901764e-01
  4.999005e-01 -6.283364e-01 -5.960645e-01                4.999005e-01 -6.283364e-01 -5.960645e-01
Eigenvalues of Saupe Tensor S(xx), S(yy), S(zz):        Eigenvalues of Alignment Tensor A(xx), A(yy), A(zz):
 -1.704800e-04 -8.877983e-04  1.058278e-03               -1.136534e-04 -5.918655e-04  7.055189e-04

Alignment Tensor Irreducible Representation (A0, A1R, A1I, A2R, A2I):
 -2.712964e-05  4.336825e-04  1.005830e-03  2.211319e-04  6.114452e-04

Tensor Properties:
A(axial)      =   1.058278e-03        # alignment tensor axial component   = 3/2*A(zz) = S(zz)
A(rhombic)    =   4.782122e-04        # alignment tensor rhombic component = A(xx) - A(yy) = 2/3*(S(xx) - S(yy))
A(rhombicity) =   4.518775e-01        # alignment tensor rhombicity        = A(rhombic) / A(axial)
A(asymmetry)  =   6.778162e-01        # alignment tensor asymmetry         = (A(xx) - A(yy))/A(zz) = (S(xx) - S(yy))/S(zz)
GDO           =   1.136428e-03        # generalized degree of order        = sqrt(3/2)*|A(xx),A(yy),A(zz)| = sqrt(2/3)*|S(xx),S(yy),S(zz)|

Results for Multi-Parameter SVD Fit of Calculated and Experimental Data:
           D(calc)[HZ]     +/- Error    D(exp)[HZ]     +/- Error  Rel. Weights            D(exp)-D(calc)        Normalized Weights    Atom Labels
D[01] =       5.211157           -       4.920000     0.500000     1.000000    r[01] =      -0.291157    w[01] =       0.041667 *) C5-H5
D[02] =      19.310048           -      19.240000     0.500000     1.000000    r[02] =      -0.070048    w[02] =       0.041667 *) C6-H6
D[03] =      12.481171           -      12.440000     0.500000     1.000000    r[03] =      -0.041171    w[03] =       0.041667 *) C7-H7
D[04] =       5.210592           -       5.260000     0.500000     1.000000    r[04] =       0.049408    w[04] =       0.041667 *) C8-H8
D[05] =      15.524370           -      15.570000     0.500000     1.000000    r[05] =       0.045630    w[05] =       0.041667 *) C10-H10A+C10-H10B
D[06] =     -18.092263           -     -18.080000     0.500000     1.000000    r[06] =       0.012263    w[06] =       0.041667 *) C11-H11A+C11-H11B
D[07] =       5.216423           -       5.250000     0.500000     1.000000    r[07] =       0.033577    w[07] =       0.041667 *) C12-H12A+C12-H12B
D[08] =     -11.848404           -     -11.890000     0.500000     1.000000    r[08] =      -0.041596    w[08] =       0.041667 *) C14-H14A+C14-H14B
D[09] =       0.889230           -       1.000000     0.500000     1.000000    r[09] =       0.110770    w[09] =       0.041667 *) C15-H15
D[10] =     -16.208762           -     -16.210000     0.500000     1.000000    r[10] =      -0.001238    w[10] =       0.041667 *) C16-H16A+C16-H16B
D[11] =     -20.380622           -     -20.390000     0.500000     1.000000    r[11] =      -0.009378    w[11] =       0.041667 *) C18-H18A+C18-H18B
D[12] =      -9.087854           -      -9.080000     0.500000     1.000000    r[12] =       0.007854    w[12] =       0.041667 *) C24-H24A+C24-H24B
D[13] =      15.231382           -      15.290000     0.500000     1.000000    r[13] =       0.058618    w[13] =       0.041667 *) C27-H27
D[14] =      14.128782           -      14.060000     0.500000     1.000000    r[14] =      -0.068782    w[14] =       0.041667 *) C30-H30
D[15] =     -11.133332           -     -11.240000     0.500000     1.000000    r[15] =      -0.106668    w[15] =       0.041667 *) C35-H35
D[16] =     -14.996828           -     -15.020000     0.500000     1.000000    r[16] =      -0.023172    w[16] =       0.041667 *) C37-H37
D[17] =       4.385002           -       4.180000     0.500000     1.000000    r[17] =      -0.205002    w[17] =       0.041667 *) C39-H39
D[18] =     -19.472174           -     -19.530000     0.500000     1.000000    r[18] =      -0.057826    w[18] =       0.041667 *) C41-H41
D[19] =      13.990533           -      13.990000     0.500000     1.000000    r[19] =      -0.000533    w[19] =       0.041667 *) C43-H43A+C43-H43B
D[20] =       2.466994           -       2.470000     0.500000     1.000000    r[20] =       0.003006    w[20] =       0.041667 *) C44-H44A+C44-H44B
D[21] =     -10.413723           -     -10.390000     0.500000     1.000000    r[21] =       0.023723    w[21] =       0.041667 *) C46-H46A+C46-H46B
D[22] =      -0.885231           -      -0.910000     0.500000     1.000000    r[22] =      -0.024769    w[22] =       0.041667 *) C47-H47
D[23] =       3.471841           -       3.530000     0.500000     1.000000    r[23] =       0.058159    w[23] =       0.041667 *) C48-H48
D[24] =      -2.419595           -      -2.450000     0.500000     1.000000    r[24] =      -0.030405    w[24] =       0.041667 *) C58-H58A+C58-H58B

Results for Multi-Parameter Fit of Calculated and Experimental Data:
rank  =              5                # rank of cosine matrix (check input if rank < 5)
cond  =   3.284159e+00                # condition number of cosine matrix (check input and singular values if very large)
chisq =       0.007595                # weighted total sum of squared residuals
aic   =      10.729085                # information criterion (AIC) for 5 degrees of freedom
rmsd  =       0.087147                # unweighted root mean square deviation
qfac  =       0.007127                # weighted Q-Factor as defined by Cornilescu
r^2   =       0.999949                # coefficient of determination r^2 = 1 - chi^2 / (weighted sum of squares)
```

Table S4. Typical ConArch$^+$/rDG input parameter file on vincristine (NOE + 3 AM RDC data sets) used similarly for all simulations reported in the main paper.

```
# Parameter options (case insensitive):
verbosity       0        # set ConArch+ verbosity
monte_carlo     off      # enable Monte-Carlo error estimates on final DG structures
constraint_rch  1.090    # constrain CH-bond length in RDC calculations
force_const     1.000    # force constant on RDC/RQC/RCSA/NOE/JHH etc.
force_c_rdc     0.500    # force constant on RDCs
#force_c_rqc    1.000    # force constant on RQCs
#force_c_csa    1.000    # force constant on RCSAs
force_c_noe    60.000    # force constant on NOEs
#force_c_jhh    0.100    # force constant on JHHs (coupling constants)
#force_c_dbl  100.000    # force constant on DBLs (double bond restraints)
#force_c_vol   10.000    # force constant on VOLs (ensemble chiral volume differences)
#force_c_tor 1000.000    # force constant on TORs (ensemble cos(torsions) differences)
force_c_mol     1.000    # force constant on molar fractions (ensembles only)
force_c_g4d    10.000    # force constant for 4D => 3D projection optimization
enable_nprocs   auto     # number of processors used for split DG jobs    (auto = no. of processors available)
enable_njobs    auto     # number of split DG jobs to be used in parallel (auto = no. of processors available)
remove_tmpfiles on       # remove some temporary files (split DG jobs only)
remove_outfiles off      # remove some output files    (split DG jobs only)
enable_parallel on       # use parallel mode for RDC/RQC/RCSA tensor calculations
enable_fastmode on       # use fast mode if not using NOE/JHH/RDC/RQC/RCSA and 4D => 3D optimization
enable_deltavol on       # use chiral volume differences for DG ensemble runs
enable_deltator on       # use cos(phi) torsion differences for DG ensemble runs
enable_aic      off      # include AIC in DG sorted output lists
enable_bic      off      # include BIC in DG sorted output lists
enable_J2D      on       # use the definition T=J+2D ('on') or T=J+D ('off') for exp. RDCs
enable_analytic on       # use analytic Cartesian gradients for RDCs
enable_3D       on       # use RDC/RQC/RCSA/NOE/JHH restraints in 3D mode
enable_4D       on       # use RDC/RQC/RCSA/NOE/JHH restraints in 4D mode
enable_G4D      on       # use 4D => 3D projection optimization and 4D gradients
enable_RDCERR   off      # use RDC/RQC/RCSA Gaussian error variates on experimental data
enable_ENERGY   on       # use energy averages
g4d_coupling    150.0    # temperature coupling of 4D => 3D projection optimization
noe_lognormal   on       # use log-normal potentials on NOEs (use harmonics otherwise)
noe_power6      on       # use r^-6 scaling for NOEs (otherwise use r^-3)
scale_grad      on       # scale RDC/RQC/RCSA/NOE/JHH gradients
scale_grad_max  250.0    # maximum scaled RDC/RQC/RCSA/NOE/JHH gradient
scale_grad_pow    2.0    # used for RDC/RQC/RCSA/NOE/JHH gradient scaling
gdesc_nsteps    5000     # DG gradient descent optimization steps
gdesc_stepinit  0.005    # DG gradient descent initial step size
gdesc_steplimit 1.00e-10 # DG gradient descent step size convergence limit
gdesc_deltaemax 1.00e-08 # DG gradient descent energies convergence limit
pop_stepsize    0.005    # initial step size on molar fractions (ensembles only)
pop_coupling    150.0    # temperature coupling of molar fractions (ensembles only)
fit_tensor_mcmt off      # use multi-conformer multi-tensor (MCMT) fitting (ensembles only)
fit_center_geom off      # use geometry-weighted fitting (ensembles only)
fit_center_mass on       # use mass-weighted fitting (ensembles only)
fit_center_hydr off      # use hydrogen atoms for fitting (ensembles only)
fit_center_iter 3        # number of iterations used for fitting (ensembles only)
fit_center_save on       # save fitted DG structures (single-conformer non-ensembles only)

# Output files:
dgfile          shake2-sorted.dgcoor3
#xyzfile        shake2-sorted.xyz
#xyzfamilies    shake2-family%02d.xyz
#pdbfile        shake2-sorted.pdb
#pdbfamilies    shake2-family%02d.pdb
#datfile4D      shake1-sorted.dat
datfile3D       shake2-sorted.dat
txtfile         shake2-bestfit.txt
inpfile         shake2-bestfit-medium%02d.inp

# Input files including exp. data:
# Reference structure:
rdcfile         vinc.inp
rdcfile         vincristine-medium01.inp
rdcfile         vincristine-medium02.inp
rdcfile         vincristine-medium03.inp

# Atomic options:
list_exclude    CH2 C42
#list_include   <set list of atom names used for configuration sorted lists here>
#list_isfixed   <set list of atom names used for configuration sorted lists here>
fit_exclude     C19 O20 O21 C22 O23 C24 C25 C33 C35 O36 O49 C50 O51 O52 C53 O54 C55 O56 C57 C58 C59
#fit_include    <set list of atom names used for fitting here (ensembles only)>
```
